# From static snapshots to longitudinal trajectories: artificial intelligence in women’s reproductive and ovarian health

**DOI:** 10.3389/fendo.2026.1893963

**Published:** 2026-08-12

**Authors:** Yuhang Liu, Yangling Liu, Yixuan Zhao, Yanfu Luo, Xiaoming Hao

**Affiliations:** 1 The Second Clinical College, Chongqing Medical University, Chongqing, China; 2 Soochow University, Suzhou, Jiangsu, China; 3 Tianjin Medical University, Tianjin, China; 4 Southwest Medical University, Luzhou, Sichuan, China; 5 The Second Affiliated Hospital of Chongqing Medical University, Chongqing, China

**Keywords:** artificial intelligence, longitudinal studies, ovarian neoplasms, ovarian reserve, PCOS

## Abstract

**Aim:**

To distinguish ovarian longitudinal biology from direct ovarian AI evidence and transferred time-series methodology, and to evaluate how temporal information is used and linked to clinical action across ovarian malignancy, PCOS, ovarian aging/POI, and ART.

**Methods:**

This structured narrative review mapped prior reviews and was supplemented by design verification of representative primary studies identified through searches covering 1 January 1989 to 1 January 2026. Two authors independently conducted the design-level verification and classification; disagreements were resolved by consensus. Evidence was classified as direct ovarian AI evidence, ovarian longitudinal biological evidence, or transferred methodological evidence. Temporal use and decision linkage were classified as T0 static prediction, T1 serial-feature summarization, T2 explicit time-aware modeling, or T3 decision-linked temporal modeling. T0–T3 describe how temporal information is used and linked to clinical decisions; they do not represent an automatic hierarchy of model quality. Recorded/calendar time, biological time, and decision time were distinguished because observation time is not necessarily equivalent to biological time.

**Results:**

Available review-level evidence and the representative primary studies verified in detail indicated that AI applications in PCOS, ovarian-cancer imaging, and ovarian-reserve assessment were predominantly T0. CA-125 velocity, half-life, and nadir represented T1 serial-feature evidence rather than AI time-series modeling. Direct T2 evidence was sparse and included a longitudinal CA-125 joint model and a small number of within-cycle ART prescription models. T3 evidence linking outputs to prespecified action thresholds, calibration, false-positive burden, net benefit, clinician override, and prospective clinical outcomes was limited. Several studies described as longitudinal AI instead established longitudinal ovarian biology or transferred temporal methods from other diseases.

**Conclusion:**

The clinical value of temporal AI in ovarian health depends less on architectural complexity than on correct alignment with ovarian biology, direct within-patient temporal evidence, calibrated validation, and a beneficial clinical action.

## 1 Introduction

The dynamic evolution of women’s reproductive and ovarian health is a core issue at the intersection of public health, reproductive endocrinology, oncology, and clinical medicine. The ovary sustains reproductive capacity and contributes to systemic endocrine and metabolic homeostasis (1). Ovarian malignancies, polycystic ovary syndrome (PCOS), and natural or pathological loss of ovarian reserve exhibit heterogeneous and nonlinear biological courses (2–7). Assisted reproductive technology (ART), however, is not a disease category; it is a treatment context in which ovarian response is repeatedly observed and acted upon. This review therefore addresses four ovarian-health clinical contexts: ovarian malignancy, PCOS, ovarian aging and premature ovarian insufficiency (POI), and ART-related ovarian response.

Clinical assessment has traditionally relied on cross-sectional biochemical indices, ultrasound images, pathology slides, or clinicopathological variables obtained at a single encounter. A single measurement cannot estimate within-person change; however, repeated measurements do not automatically produce accurate individual trajectories or beneficial clinical decisions. For AMH, the prospective Doetinchem cohort found that adding the individual decline rate did not improve menopause prediction beyond age 25 years and generally underestimated early-menopause risk (8). For ovarian cancer, the MRC OV05/EORTC 55955 randomized trial showed that treatment initiated earlier solely because CA-125 had risen did not improve overall survival compared with treatment delayed until clinical or symptomatic relapse (9). These examples separate earlier observation from validated individual prediction and from beneficial intervention.

Longitudinal data can support estimation of change, latent state, and treatment response, but only when measurement timing, missingness, and care processes are modeled explicitly. Electronic health records, repeated biomarkers, serial imaging, and cycle-level monitoring create opportunities for patient-level updating, whereas static multimodal fusion remains static when its inputs represent only one encounter or a fixed short window. Recurrent networks, Transformers, survival models, and missingness-aware methods are relevant tools, but architectural complexity alone does not establish longitudinal evidence. A model is explicitly time-aware only when it uses within-patient order, irregular intervals, time-varying covariates, missingness patterns, latent states, or risk updates as new data arrive (10–17).

To prevent biological follow-up from being conflated with implemented temporal AI, this review distinguishes three evidence sources. Direct ovarian AI evidence comprises models developed and evaluated in ovarian-health populations for the stated task. Ovarian longitudinal biological evidence comprises cohort, functional-data, or biomarker studies that establish within-person or population-level biological change but do not themselves demonstrate an AI system for dynamic management. Transferred methodological evidence comprises time-series methods validated in other organs, diseases, or general EHR populations and considered only as methodological analogues. Representative primary studies were checked at the design level before being assigned to one of these evidence sources.

**Figure 1:**
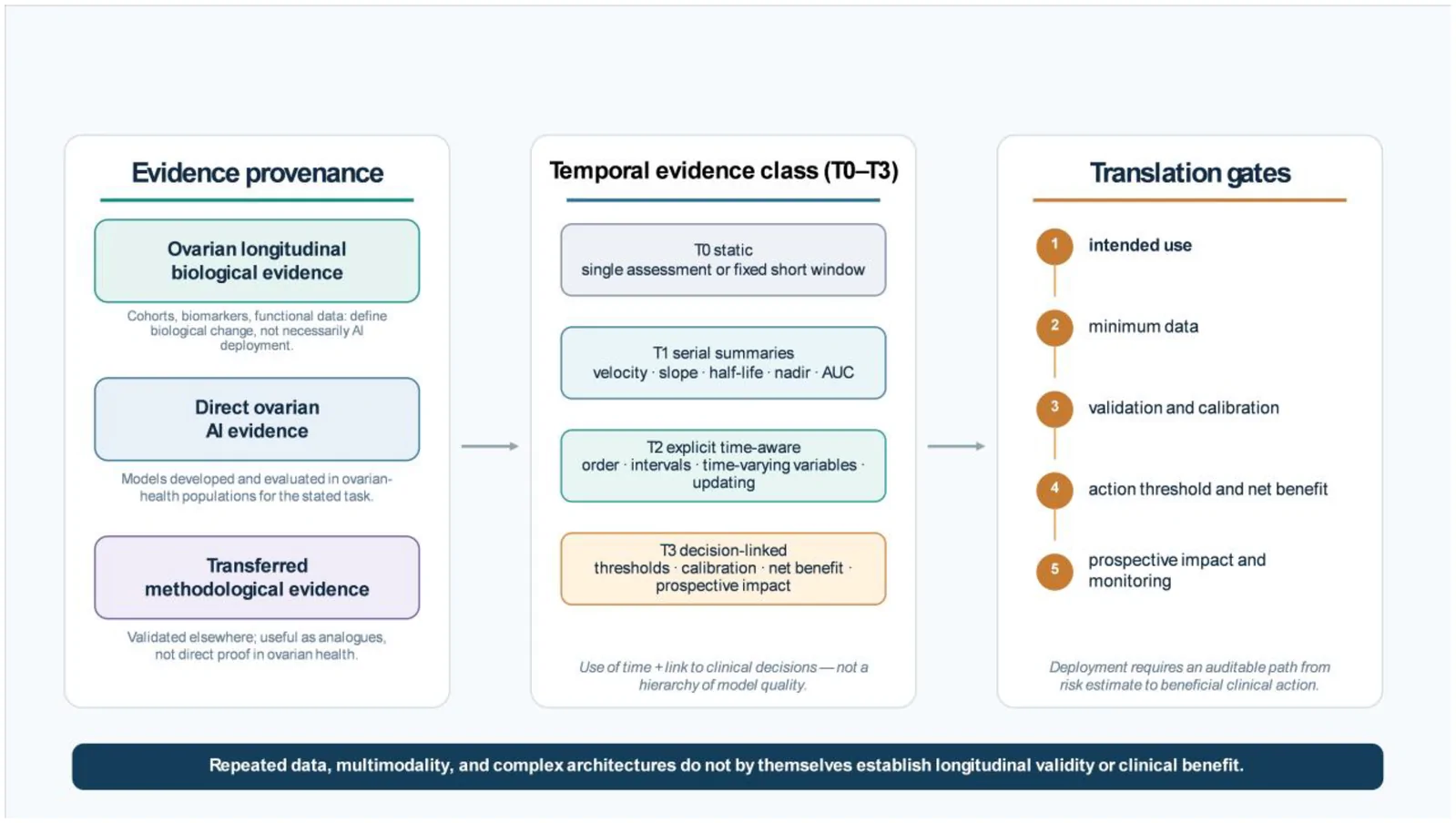
Evidence provenance, temporal-information use, and clinical actionability in ovarian-health AI. The framework separates evidence source, temporal representation, and decision-linked translation; T0–T3 are author-defined temporal evidence classes and do not represent a hierarchy of model quality.

Across these evidence sources, the manuscript uses one formal classification based on temporal-information use and connection to clinical decisions. T0, static prediction, uses a single time point or fixed short window, including single-time-point multimodal data. T1, serial feature summarization, compresses repeated observations into velocity, slope, half-life, nadir, change score, or area under the curve. T2, explicit time-aware modeling, uses within-patient order, irregular intervals, time-varying covariates, missingness patterns, latent states, or risk updating when new observations arrive. T3, decision-linked temporal modeling, connects model outputs to predefined clinical actions and evaluates action thresholds, calibration, false-positive burden, net benefit, human override, or prospective clinical impact. T0–T3 describe how temporal information is used and linked to clinical decisions; they do not represent an automatic hierarchy of model quality. The relationship among evidence provenance, temporal-information use, and decision-linked translation is summarized in **Figure 1**.

Recorded/calendar time, biological time, and decision time are used as interpretive concepts rather than additional grades. Observation time is not necessarily equivalent to biological time. A PCOS clinic visit does not necessarily identify menstrual-cycle phase; chronological age does not specify an individual’s ovarian-reserve state; the same ART stimulation day does not imply the same follicular-development stage; and the same month of ovarian-cancer follow-up does not imply the same tumor state. Decision time adds a further distinction: a detectable change can precede the point at which acting on that change produces net clinical benefit.

Previous reviews have already summarized AI applications in ovarian cancer, PCOS, ovarian reserve assessment, and ART, and have discussed multimodal fusion and time-series learning (15, 18–22). The PCOS systematic review included 31 diagnostic and classification studies, all observational and based on retrospective samples, while a comprehensive ART systematic review analyzed 48 articles (20, 22). The novelty of the present review therefore cannot rest on covering more application areas. The unresolved gap is not another application inventory, but a cross-context analysis that distinguishes biological longitudinal evidence, direct time-aware ovarian AI, and transferred methodology, and evaluates whether model outputs are connected to beneficial clinical actions.

Accordingly, this structured narrative review critically evaluates the four ovarian-health clinical contexts through the T0–T3 framework, supplemented by study-design verification of representative original investigations. It asks where ovarian longitudinal biology is established, where direct within-patient temporal AI has actually been implemented, where methods are merely transferred from other domains, and whether any temporal output has been validated against calibrated action thresholds and clinically meaningful outcomes.

## 2 Evidence acquisition, verification, and analytical classification

This structured narrative review was informed by two complementary search streams in PubMed/MEDLINE and Web of Science Core Collection. A temporal-evidence stream combined ovarian-health terms with longitudinal, time-series, repeated-measurement, and dynamic-prediction terms and with AI or relevant statistical-modeling terms. A second domain-specific AI stream omitted the temporal-term requirement and was used to identify static or cross-sectional AI studies and prior reviews that could otherwise be missed by a time-restricted query. Searches were supplemented by targeted checks of arXiv and online-ahead-of-print sections of relevant medical and computational journals. Search concepts included ovarian malignancy, PCOS, ovarian reserve, ovarian aging, premature or primary ovarian insufficiency, ART, missingness-aware learning, multimodal fusion, time-series modeling, Transformers, and large language models. Searches covered publications and publicly available preprints from 1 January 1989 through 1 January 2026, including articles available online ahead of issue publication by the cutoff date. Detailed search strings are provided in **Supplementary Methods S1**. Where a preprint publicly available by the cutoff date subsequently appeared as a peer-reviewed article, the citation was updated to the version of record without changing the evidence set.

### 2.1 Review-level evidence mapping

Separate review-level searches were conducted for ovarian cancer AI reviews, PCOS AI reviews, ovarian reserve/aging AI reviews, ART AI reviews, reproductive medicine LLM reviews, and longitudinal clinical AI reviews. These reviews were used to map the existing evidence landscape, identify duplicated claims and previously covered methodological themes, and locate representative primary studies for design-level verification. Review conclusions were not treated as direct evidence that time-aware AI had been implemented in an ovarian-health population. The scope and analytical coverage of representative previous reviews are compared in **Supplementary Table 1**.

### 2.2 Representative-study verification

For each core study supporting a major claim, the full report was checked for population, study design, repeated within-patient measurements, temporal representation, outcome, comparator, validation, and clinical action. Two authors independently conducted the design-level verification and classification; disagreements were resolved by consensus. Classification was based on the actual data structure and analytic procedure rather than on terms such as “dynamic,” “longitudinal,” or “trajectory” in the title or discussion. Studies using repeated observations only to derive a slope, velocity, half-life, nadir, change score, or area under the curve were distinguished from models that explicitly processed ordered patient-level sequences or updated risk when new observations became available. The resulting design-level evidence matrix is provided in **Supplementary Table 2**.

### 2.3 Evidence classification

Studies were categorized as direct ovarian AI evidence, ovarian longitudinal biological evidence, or transferred methodological evidence. They were further classified as T0, T1, T2, or T3 according to the use of temporal information and linkage to clinical decisions. T0 covered static prediction from a single assessment or fixed short window, including single-time-point multimodal data. T1 covered serial-feature summarization. T2 required explicit use of within-patient order, timing, time-varying covariates, missingness patterns, latent states, or risk updating. T3 required linkage to a prespecified clinical action and evaluation of action thresholds, calibration, false-positive burden, net benefit, clinician override, or prospective clinical impact. T0–T3 describe how temporal information is used and linked to clinical decisions; they do not represent an automatic hierarchy of model quality. Recorded/calendar time, biological time, and decision time were used as interpretive concepts, not additional grades. Observation time is not necessarily equivalent to biological time. The biological temporal structures, evidence status, and translation requirements across ovarian-health contexts, together with a comparison of reserve-modeling designs, are summarized in **Tables 1**, **2**.

**Table 1:** Biological temporal structure, evidence status, and translation requirements across ovarian-health contexts.

Clinical context	Biological temporal structure	Evidence status	Typical temporal evidence class	Core minimum inputs	Appropriate target	Potential action	Main barrier
Ovarian malignancy	Event-risk process perturbed by surgery, chemotherapy, maintenance therapy, occult recurrence, and irregular symptom- or marker-triggered observation.	Limited. CA-125 velocity, half-life, and nadir are T1 summaries; direct T2 updating is sparse; pathology and radiomics evidence is predominantly static; serial imaging evidence is transferred from lung cancer (31–33, 37, 39–48).	Predominantly T0-T1; few T2 candidates; no established T3 identified.	Exact dates of surgery, systemic and maintenance therapy, CA-125, and imaging; stage and residual disease; symptoms; assay and imaging protocol.	Calibrated recurrence or event risk updated after new measurements and conditional on treatment history.	Repeat biomarker testing, additional imaging, shorter follow-up, clinical-trial referral; treatment only under a validated rule.	Informative observation and treatment confounding; no universal action threshold; earlier CA-125-triggered treatment did not improve survival (9).
PCOS	Phase-dependent, state-switching, multiscale endocrine-metabolic process across cycles, reproductive stages, and years.	Predominantly T0 diagnosis or classification. Longitudinal cohorts characterize obesity and insulin-resistance biology but do not demonstrate deployed dynamic AI (20, 49–51).	Predominantly T0; T2 and T3 evidence is very limited.	Exact timestamps; age and reproductive stage; menstrual pattern or phase; ovulatory status; androgens; BMI and waist; OGTT, glucose or HbA1c; lipids; medications; pregnancy intention.	Updated metabolic or ovulatory risk across life stages, rather than repeated binary diagnosis.	Metabolic testing and follow-up, liver-risk assessment, lifestyle support, and ovulation-related management.	Biological phase is often unrecorded; phenotypes and care pathways are heterogeneous; no validated dynamic action thresholds (52).
Ovarian aging	Latent nonlinear reserve decline with substantial between-woman heterogeneity, assay noise, short-term variation, and lower detection limits.	Longitudinal biological and statistical evidence exists, but the gain from repeated AMH is inconsistent; OvaRePred and retinal age gap are trajectory-inspired or cross-sectional, not within-woman T2 models (8, 36, 53–56).	T0-T1; direct T2 and T3 not established.	Age; assay-harmonized AMH dates and values; AFC, FSH, and estradiol when relevant; reproductive history; hormonal exposures; assay platform.	Calibrated latent-reserve or event risk with prediction intervals, not an exact individual menopause date.	Risk communication, repeat testing when indicated, specialist referral, and fertility-preservation counselling.	Cohort-, assay-, age-, and model-dependence; censoring, extrapolation, and wide individual uncertainty.
POI	Heterogeneous boundary condition before age 40; genetic, iatrogenic, autoimmune, or unexplained causes; intermittent ovarian activity may occur.	Direct longitudinal AI evidence was not identified among the review-level evidence mapped and representative primary studies verified. AMH is neither the primary diagnostic test nor recommended for routine POI prediction (57).	Limited to T0 risk identification; T2 and T3 evidence was not identified in the reviewed evidence.	Age; menstrual history; pregnancy status; FSH with repeat testing when needed; estradiol; gonadotoxic exposure; genetic and autoimmune history; AMH only for residual diagnostic uncertainty.	Identify high-risk iatrogenic cases or unexpectedly rapid decline and prompt guideline-based evaluation, not diagnose POI from low AMH.	Specialist evaluation, etiologic work-up, fertility-preservation counselling, and long-term endocrine, bone, and cardiovascular care.	Rare heterogeneous outcomes, intermittent activity, sparse labeled longitudinal cohorts, and diagnosis governed by clinical criteria.
ART	Short-interval treatment-response feedback: prior dose changes later hormones and follicles, which alter monitoring and the next decision.	Daily FSH prediction is retrospective single-center T2; prospective dose and trigger support is an early T3 candidate; automated follicle counting standardizes measurement only (34, 35, 58).	T2 candidates; early T3 not conclusive.	Exact stimulation day and time; prior doses; protocol; estradiol, LH, and progesterone; follicle sizes and counts; age, BMI, AMH and AFC; prior response; clinician actions.	Clinician-facing response prediction or a causal treatment policy linked to mature-oocyte, safety, and live-birth outcomes, not imitation of historical prescriptions.	Dose adjustment, monitoring interval, and trigger or retrieval planning, with clinician override.	Time-varying confounding; behavior labels are not optimal-treatment labels; limited external and prospective patient-outcome evidence.

**Table 2:** Static reserve classification, trajectory-inspired endocrine-age mapping, and true within-woman longitudinal modeling.

Comparison domain	Bologna/POSEIDON	OvaRePred	True within-woman longitudinal model
Data design	Consensus or rule-based ART classification using age, ovarian-reserve tests, and prior stimulation response (91, 92).	Single-center retrospective ART-cycle data; one analyzed cycle per woman; temporally separated same-center training and test periods (36).	A longitudinal cohort or registry with repeated observations from the same woman, linked event and treatment histories, and prespecified follow-up.
Repeated within-woman measurements	Not required. A previous stimulation response may inform classification, but serial biomarkers are not modeled as a trajectory.	No long-term repeated AMH series from the same woman; repeated cycles from the same woman were removed from analysis.	Required for the longitudinal target: multiple dated measurements per woman, with the number and timing of observations explicitly represented.
Use of intervals	No explicit use of elapsed time or irregular observation intervals.	No woman-specific measurement intervals. Future milestones rely on a fixed-interval assumption applied to a population curve.	Explicitly uses age, cycle day, time since prior observation or exposure, and potentially irregular intervals and informative visit timing.
Individual slope	Not estimated.	Not estimated. A current POR probability is mapped onto a population curve rather than used to infer a woman-specific decline rate.	Estimated directly or represented through random effects, latent states, change points, or sequential risk updating, with uncertainty propagated.
Measurement-error modeling	Not part of the classification rules.	Conventional regression and calibration; no explicit latent-process model separating AMH measurement error from underlying reserve.	Separates observed values from a latent biological process using mixed-effects, hierarchical Bayesian, state-space, or related measurement-error models.
Output	A categorical poor-response or low-prognosis group for ART stratification.	POR probability, ovarian-reserve score, endocrine-age mapping, and hypothesis-generating milestone projections.	An updated latent-reserve trajectory, woman-specific rate of change, event risk, or decision-relevant forecast after each new observation.
Uncertainty	Usually categorical; does not provide an individualized trajectory interval.	Model discrimination and calibration are reported, but milestone projections do not quantify full individual trajectory uncertainty.	Reports prediction intervals, posterior or confidence intervals, calibration, and uncertainty in latent state, slope, event risk, and decision threshold.
Validation	Consensus reproducibility and prognostic stratification; not validation of a longitudinal prediction model.	Same-center temporal testing and limited population comparison; prospective longitudinal validation in non-ART cohorts remains necessary.	Requires external and temporal validation, assay and population transportability assessment, and ideally prospective evaluation of clinical impact.
Main limitation	Static or episode-based categories cannot estimate within-woman change and may combine heterogeneous mechanisms within the same group.	Trajectory language exceeds the data structure: ART selection, population mapping, and the fixed-interval assumption limit individual longitudinal claims.	Data-intensive and vulnerable to assay drift, informative observation, missingness, treatment confounding, censoring, and poor transportability.

Because this was a structured narrative review rather than a systematic review or meta-analysis, performance metrics from heterogeneous tasks and populations were not quantitatively pooled or directly ranked.

## 3 Defining temporal evidence in ovarian-health AI

Temporal evidence should be defined by the information actually supplied to a model and by the decision process it supports, not by the sophistication of its architecture. Recurrent neural networks can represent ordered observations and Transformers can model long-range dependencies, irregular event sequences, and high-dimensional clinical histories when time stamps, masks, or interval encodings are explicitly incorporated (16, 23–28). These capabilities are useful, but neither architecture guarantees that a study contains repeated within-patient data, represents biological change, or updates a clinical decision. A Transformer is not a longitudinal model merely because it can process sequences.

Multimodality is likewise distinct from temporality (15, 29). Combining a clinical table with one ultrasound examination, one pathology slide, or one preoperative CT scan may improve static prediction but remains a single-time-point analysis. Conversely, a modest statistical model can be genuinely longitudinal if it estimates within-person change or links a repeatedly measured biomarker to an event process. Image encoders, RNNs, Transformers, and fusion layers should therefore be treated as implementation choices rather than evidence grades. The primary questions are whether measurements are repeated within the same patient, how their ordering and spacing are represented, whether the model is updated when new data arrive, and whether its output changes a prespecified clinical action.

### 3.1 Static, serial, time-aware, and decision-linked models

T0—Static prediction. T0 models use a single assessment or a fixed short observation window and do not estimate a patient-specific temporal process. Single-time-point multimodal models are still T0. For example, a PCOS classifier combining contemporaneous clinical variables and ovarian ultrasound is a T0 static multimodal model, even if it uses deep learning or cross-attention (20). The platinum-resistance recurrence model (30) is also T0 because it uses perioperative clinical, pathological, and laboratory variables in a retrospective single-center classification analysis rather than an ordered within-patient sequence.

T1—Serial feature summarization. T1 studies contain repeated measurements, but the temporal record is compressed into summary quantities such as velocity, slope, half-life, nadir, change score, or area under the curve. CA-125 velocity, elimination half-life, and nadir are therefore T1 serial-feature evidence rather than AI time-series modeling (31–33). The PLCO study (32) is specifically a screening analysis of subsequent ovarian-cancer occurrence; it should not be presented as evidence that CA-125 velocity has been validated for post-treatment recurrence monitoring.

T2—Explicit time-aware modeling. T2 requires the model to use at least one substantive temporal property, including the ordered sequence of measurements, irregular intervals, time-varying covariates, missingness patterns, latent states, or risk updating after new observations. The daily FSH model (34) is a T2 time-aware prescription-prediction model because it processes serial monitoring information across stimulation days. However, its retrospective single-center target was the historical prescribed dose, so it does not establish a biologically optimal dose or a closed-loop treatment policy.

T3—Decision-linked temporal modeling. T3 connects a time-aware output to a prespecified clinical action and evaluates the consequences of acting on it. Relevant evidence includes action thresholds, calibration at the decision point, false-positive burden, decision-curve or net-benefit analysis, clinician override, safety, and prospective clinical impact. A model evaluated in a prospective intervention study with predefined treatment rules and demonstrated clinical net benefit would meet T3 criteria. Current ovarian-health evidence rarely satisfies this full standard; the prospective ART study (35) is better regarded as an early T3 candidate than as thorough validation because key differences in mature oocyte yield and total FSH were not statistically significant.

OvaRePred illustrates why terminology alone is insufficient. It predicts poor ovarian response from age and AMH in retrospective ART-cycle data and maps the resulting probability onto a population ovarian-aging curve to derive an endocrine age (36). It is therefore a trajectory-inspired T0 cross-sectional endocrine-age mapping, not a T2 model of repeated within-woman ovarian decline. T0–T3 describe how temporal information is used and linked to clinical decisions; they do not represent an automatic hierarchy of model quality. .

### 3.2 Recorded time is not biological time

PCOS. Clinic dates do not necessarily identify menstrual-cycle phase, ovulatory status, medication exposure, or a stable endocrine state. PCOS also evolves over several time scales: hormone concentrations may vary within a cycle, ovulatory states may transition across months, and metabolic risk may accumulate over years. A model based only on visit order can therefore confound care-seeking patterns with biological transitions unless cycle phase and relevant state variables are represented.

Ovarian aging and POI. Chronological age is recorded calendar time, whereas ovarian reserve is a latent biological state with nonlinear decline, substantial between-woman heterogeneity, assay variation, and short-term biological noise. Repeated AMH measurements can help characterize population patterns, but they do not automatically recover an accurate individual trajectory, as shown by inconsistent gains in menopause prediction across cohorts (8, 53). Models should separate the latent decline process from measurement error and should quantify uncertainty around individual extrapolation.

ART-related ovarian response. Stimulation day is not equivalent to a common follicular-development stage across patients. Gonadotropin dose affects subsequent hormone levels and follicle growth, which then influence the next dose, creating a treatment–response feedback loop. A time-aware ART model must distinguish untreated prognosis from treatment-mediated response and must account for the fact that physician actions are part of the observed sequence rather than neutral measurements.

Ovarian malignancy. Months since surgery or chemotherapy do not place all patients in the same tumor state. Cytoreduction, chemotherapy, maintenance treatment, treatment discontinuation, and occult recurrence perturb biomarker and imaging trajectories, while the event process includes censoring and potentially competing outcomes. Longitudinal marker modeling should therefore be aligned with recurrence or survival risk and with the intervention that would follow a risk update, rather than treating follow-up time as a direct proxy for tumor evolution.

Observation time is not necessarily equivalent to biological time. Recorded/calendar time indicates when data were obtained; biological time describes the latent disease or reserve state; decision time identifies when a clinical action is available and potentially beneficial. These concepts assist interpretation but do not constitute additional evidence grades.

### 3.3 Model selection should follow the clinical question

Model choice should begin with the estimand and clinical workflow. For repeated AMH measurements, nonlinear mixed-effects models are well suited to estimating a population decline function while allowing woman-specific random effects and separating between-person heterogeneity from within-person error (53). For CA-125 followed until recurrence or death, joint longitudinal–survival models can link the latent marker trajectory to an event hazard and update risk as new values become available; an ovarian-cancer study has applied this approach directly to longitudinal CA-125 and recurrence (48). State-space models are appropriate when the target is an unobserved biological state that evolves over time and is measured with noise, and they can be extended to survival prediction (59).

ART poses a different question because treatment decisions alter subsequent observations. Causal longitudinal methods and dynamic treatment regimes are more appropriate when the objective is to estimate which dose or action should be chosen at each decision point to improve a clinical outcome, rather than to reproduce historical prescribing (60). Such analyses require explicit treatment histories, sequential exchangeability assumptions or randomized decision data, clinically meaningful utility functions, and policy-level evaluation.

RNNs and Transformers may be justified when the sample size, sequence length, irregularity, and feature dimensionality exceed what simpler models can represent reliably. They should be compared with transparent statistical baselines and evaluated for calibration, temporal transportability, and added decision value, not only discrimination. Multimodal fusion is warranted when modalities provide complementary information collected at clinically aligned times; adding modalities without temporal alignment can increase complexity without resolving the biological question.

Attention weights, Grad-CAM, and SHAP support model inspection but do not establish causal mechanisms or sufficient clinical interpretability. Attention distributions can change without corresponding changes in predictions, and visually plausible saliency maps may be insensitive to learned model parameters or the data-generating relationship (38, 61–63). Clinical interpretability additionally requires a stable target, a transparent action pathway, calibrated uncertainty, and evidence that acting on the explanation or prediction improves patient outcomes.

## 4 Missingness and observation-process bias in ovarian longitudinal data

Missing data in ovarian-health records are not merely empty cells. Complete-case analysis can reduce sample size, alter the clinical composition of the analyzed cohort, and introduce selection bias when inclusion depends on whether a test or visit was recorded (64, 65). Last observation carried forward (LOCF) can impose biologically implausible stability, whereas multiple imputation by chained equations (MICE) depends on correctly specified models and assumptions about the missing-data mechanism (66–68). These limitations are particularly consequential for endocrine markers and treatment-response variables that may change nonlinearly or around clinically important thresholds. A numerically plausible imputation may therefore smooth an inflection point, reduce within-person variance, or create an artificial trajectory that was never observed.

Imputation error can subsequently be treated as true signal by a predictive model and may be amplified under external distribution shift (64, 69). End-to-end approaches can instead supply the model with observed-value masks, elapsed time since the previous measurement, or learned decay terms, and some architectures jointly estimate missing values and the prediction target (16, 70). These methods avoid treating all unobserved values as interchangeable, but they do not remove the need to specify why, when, and by whom observations were generated. A model may obtain high internal performance by exploiting testing frequency or visit patterns rather than ovarian biology (71–73).

### 4.1 Biological-state missingness

In ovarian-health data, the clinically relevant missing variable may be the biological state that gives meaning to a recorded measurement. In PCOS, a hormone or ultrasound result may be difficult to interpret when menstrual-cycle day, ovulatory status, recent hormonal treatment, or the relation of sampling to a state transition is absent; regular calendar cycles do not guarantee ovulation, and AMH itself may vary across the menstrual cycle (52). In ovarian aging and POI research, missing information on treatment exposure, assay platform, or reproductive state can similarly confound apparent change. During ART, a laboratory value without the corresponding stimulation day, medication history, or follicular-development stage is not simply an incomplete time series. Numeric imputation cannot reconstruct an unrecorded biological phase, so models should represent state uncertainty explicitly or restrict inference to clinically aligned observations.

### 4.2 Clinician-driven observation

Observation schedules are often adaptive consequences of prior measurements and clinical judgment. During controlled ovarian stimulation, the timing of the next estradiol test or ultrasound examination, and sometimes the dose subsequently prescribed, is influenced by the preceding follicle and hormone response (34, 74). The observation process is therefore embedded in a treatment-response feedback loop: prior state influences monitoring, monitoring influences treatment, and treatment alters the next state. In ovarian-cancer surveillance, cross-sectional imaging may be ordered because of symptoms, examination findings, or a change in CA-125 rather than at uniformly scheduled intervals (75–77). A model that treats these visits as passively sampled time points may mistake clinician concern or local surveillance policy for latent disease progression. Evaluation should therefore include observation intensity, ordering indications, treatment changes, and center-specific protocols where available.

### 4.3 Access-driven missingness

Data density also reflects who can enter and remain in care. Visit frequency and completion of laboratory or imaging follow-up may encode healthcare access, out-of-pocket cost, fertility intentions, travel distance, regional resource availability, and the workflow capacity of individual centers (78–81). For example, a patient pursuing pregnancy may undergo intensive endocrine and ultrasound monitoring, whereas a patient with similar biology but no immediate fertility intention may have sparse records. Rural or lower-resource settings may record fewer imaging studies or specialist visits even when clinical need is comparable. Consequently, missing measurements can become a proxy for socioeconomic position or institutional infrastructure, and models trained on such patterns may underperform or systematically mis calibrate risk when transported to another population or care pathway.

Missingness indicators and elapsed-time variables should therefore be analyzed as components of the observation process, not accepted uncritically as biological predictors. Development reports should describe the expected sampling schedule, deviations from that schedule, reasons for testing when available, missingness by site and patient subgroup, and performance after temporal and external validation. Sensitivity analyses should compare models with and without observation-process features and assess whether apparent gains persist across centers with different monitoring policies. Missingness patterns may improve prediction within a specific care system, but may simultaneously encode clinician behavior, access to care, and institutional workflow, thereby limiting transportability and fairness.

## 5 From serial biomarkers to decision-linked recurrence assessment in ovarian malignancies

Ovarian malignancies, particularly high-grade serous ovarian carcinoma, frequently present at an advanced stage and recur after initial treatment (82–84). Recurrence and platinum resistance arise through time-dependent interactions among residual disease, tumor evolution, treatment exposure, and the tumor microenvironment (85, 86). Nevertheless, biological dynamism does not mean that the available computational evidence is itself longitudinal. Across this literature, single measurements and single-time-point multimodal inputs remain T0; summary features derived from repeated CA-125 measurements are T1; models that explicitly update recurrence risk from ordered measurements are T2; and only models that connect an updated risk to a prespecified action and evaluate clinical consequences qualify as T3.

### 5.1 CA-125 across T0–T3: from single values to actionable risk updates

A single CA-125 concentration obtained at diagnosis or during surveillance is a T0 predictor because it provides no estimate of within-patient change. Threshold-based interpretation remains clinically familiar, but an isolated value cannot distinguish a stable patient-specific baseline from an accelerating rise, and its meaning depends on treatment phase, residual disease, assay context, and the reason for testing (75, 76). A T0 model may combine CA-125 with stage, pathology, laboratory findings, or treatment variables, but the presence of multiple variables does not make the model time-aware.

Velocity, slope, elimination half-life, nadir, time to nadir, and change scores summarize repeated CA-125 values into one or several derived features and are therefore T1 serial-feature evidence. Studies of dynamic change, half-life, or nadir have mainly used conventional regression or survival analyses to associate these summaries with progression or survival; they should not be described as AI time-series models (31, 33). The CA-125 velocity study was performed in the PLCO screening cohort and predicted incident ovarian cancer rather than post-treatment recurrence. It included 28,038 women who had at least two CA-125 screening tests, of whom 72 developed ovarian cancer, and used the compressed velocity feature in a multivariable prediction model (32). Its findings illustrate T1 feature construction in a screening setting but cannot be directly extrapolated to recurrence surveillance after treatment.

Improved temporal discrimination should not be equated with benefit from earlier treatment. In MRC OV05/EORTC 55955, 529 women in complete remission after first-line therapy were randomized to start treatment when CA-125 rose or to delay treatment until clinical or symptomatic relapse. Earlier treatment produced no overall-survival advantage compared with delayed treatment (9). This trial establishes an essential decision-time constraint: detecting a biomarker change earlier is clinically useful only when a corresponding intervention improves outcomes or reduces burden.

T2 assessment requires the model to retain the ordered within-patient measurements rather than collapse them into a single summary. Joint longitudinal–survival models can estimate a latent CA-125 trajectory together with the hazard of recurrence and update risk when a new result becomes available; a direct ovarian-cancer example has linked longitudinal CA-125 to recurrence risk (48). Dynamic landmark models offer another candidate approach by repeatedly redefining the prediction origin at clinically relevant follow-up times (87). These methods can provide patient-specific risk updates, but they remain T2 unless the update is prospectively connected to an action rule and evaluated beyond discrimination.

A representative T0 study used clinicopathological, laboratory, surgical, and chemotherapy variables to classify platinum-resistant recurrence, but did not model within-patient biomarker sequences. In that retrospective single-hospital cohort (30), CA-125, complete blood count, lactate dehydrogenase, and other laboratory measurements were primarily obtained within one week before surgery. Model performance was assessed by five-fold internal cross-validation without external validation. The study therefore provides static risk-classification evidence, not evidence of explicit temporal modeling.

At T3, a temporally updated probability must be tied to a prespecified clinical action and evaluated for calibration, false-positive burden, net benefit, clinician override, and prospective clinical impact. For example, a model might trigger confirmatory testing rather than immediate treatment at one threshold and recommend a multidisciplinary review at another. Direct ovarian evidence meeting this decision-linked standard remains limited, and the MRC trial cautions against treating a biomarker-triggered action as beneficial merely because it occurs earlier.

### 5.2 Static imaging evidence and transferred serial-imaging methodology

The ovarian pathology and imaging studies cited in this review are best characterized as static pathology, radiomics, or single-time-point multimodal prognostic evidence (37, 39–46). Whole-slide-image models have associated morphology with platinum response or treatment effectiveness, sometimes combining slide-derived representations with clinical variables (39–42). CT radiomics and deep-learning studies have used preoperative images to predict residual tumor, early disease progression, recurrence, progression-free survival, or overall survival (37, 43, 44), while PET/CT and MRI studies have predicted progression-free survival or platinum sensitivity from imaging acquired at a defined assessment point (45, 46). These studies may be clinically informative T0 models, including T0 static multimodal models, but they do not demonstrate explicit temporal integration of serial ovarian-cancer images.

The fact that CT, PET/CT, or MRI can be repeated in clinical practice does not change the evidence grade of a study that analyzes one scan per patient. Claims about temporal evolution require repeated same-patient images, preserved scan order and intervals, explicit handling of treatment between scans, and evaluation of how a new scan updates risk. Without these elements, a prognostic imaging model remains static even when its outcome is recurrence or survival.

Serial imaging in lung cancer provides transferred methodological evidence rather than direct validation in ovarian cancer. A transferred serial-imaging study in lung cancer integrated pretreatment and follow-up CT scans from patients with non-small-cell lung cancer using convolutional and recurrent components, with predictive performance improving as additional scans were incorporated (47). The design demonstrates how serial images can be modeled, but ovarian-specific validation would still need to address different treatment pathways, disease distribution, scan indications, and clinically relevant actions.

### 5.3 The action gap after an updated recurrence risk

When a biomarker or imaging-based risk estimate rises, possible actions include repeat biomarker testing, additional imaging, shortened follow-up, referral to a clinical trial, or treatment initiation. These actions have different costs, delays, false-positive consequences, and potential benefits; they should not share a single undifferentiated threshold. A useful model must specify which action is being considered, at what decision time, for which patient group, and against which comparator.

Current evidence does not establish a universal threshold at which an AI-updated recurrence risk should trigger treatment. Future T3 studies should prospectively compare decision rules, report calibration and net benefit across clinically plausible thresholds, quantify additional tests and imaging, document clinician override, and evaluate patient-centered and survival outcomes. The objective is not simply earlier detection, but a risk update that leads to a beneficial clinical action.

## 6 Static diagnostic AI and multiscale biological trajectories in PCOS

PCOS is a heterogeneous, lifelong endocrine-metabolic condition whose reproductive, metabolic, cardiovascular, and psychological manifestations vary across the life course. Consistent with the 2023 International Evidence-based Guideline, diagnosis is age- and context-dependent: in adults, it is based on combinations of ovulatory dysfunction, clinical or biochemical hyperandrogenism, and polycystic ovarian morphology, with serum AMH permitted as an alternative to ultrasound for defining polycystic ovarian morphology in appropriately selected adults; ultrasound and AMH are not recommended for diagnosis during adolescence (4, 5, 52, 88, 89). PCOS should be modeled as a phase-dependent, state-switching, and multiscale endocrine-metabolic process rather than as a simple periodic trajectory. The evidence must therefore distinguish static diagnostic AI from longitudinal biological studies that describe what a future time-aware model should represent.

### 6.1 Direct PCOS AI evidence is predominantly T0

The clearest direct PCOS AI evidence concerns diagnosis or classification from feature sets assembled at one encounter or within a fixed assessment window. A systematic review included 31 studies, all observational and retrospective, that used clinical records, ultrasound, genetic or proteomic data, or combinations of these sources. Seventeen studies used clinical information with or without imaging. Support vector machines, k-nearest-neighbor methods, and regression models were the most frequent approaches, rather than models that updated an individual patient’s risk as ordered measurements accumulated (20).

Accordingly, a model that combines one-time clinical variables with an ultrasound examination is T0 static multimodal evidence, even when it uses a complex classifier or achieves high diagnostic discrimination. It does not become longitudinal because menstrual history summarizes prior events or because several modalities are fused. The relevant review also identified heterogeneous diagnostic standards, predominantly retrospective data, limited independent validation, and high risk of bias (20). Direct PCOS AI evidence therefore remains centered on T0 diagnosis and classification; it does not yet demonstrate routine within-patient dynamic risk updating or decision-linked management.

### 6.2 PCOS longitudinal biological evidence

Longitudinal biological evidence establishes that PCOS-related risk evolves across different time scales. In the Northern Finland Birth Cohort, earlier childhood adiposity rebound and BMI trajectories from birth to mid-adulthood were associated with later PCOS diagnosis and obesity (49). During adolescence, the coexistence of PCOS and fatty liver disease was associated with a more adverse metabolic phenotype, illustrating that manifestations differ by life stage and comorbidity (50). In the Tehran Lipid and Glucose Study, functional data analysis showed distinct long-term insulin-resistance trajectories: worsening was concentrated among women with PCOS and obesity, whereas trajectories in women without obesity differed, demonstrating effect modification by BMI and waist status (51).

These studies define the biological processes that future AI should model, but they do not themselves demonstrate deployed longitudinal AI. They describe childhood-to-adult adiposity, life-stage-specific metabolic states, and repeated insulin-resistance patterns; they do not evaluate a model that prospectively updates an individual’s risk after each new measurement, selects a clinical action, or improves patient outcomes. Future T2 models should represent transitions among reproductive and metabolic states rather than assuming a smooth, uniform cycle or a single monotonic disease trajectory.

### 6.3 Clinical and temporal inputs should reflect guideline-defined care

A clinically credible PCOS trajectory dataset should include age and reproductive stage; menstrual pattern; ovulatory status when available; androgen-related indicators; BMI and waist circumference; glucose, HbA1c, or oral-glucose-tolerance-test data; lipids; medication exposure; pregnancy intention; and exact timestamps. Exact time should be accompanied by biological context, including cycle phase when known, hormonal treatment, pregnancy or postpartum status, and major weight or lifestyle changes. Without these variables, a model may confuse a change in testing context with a change in endocrine-metabolic state.

The 2023 international guideline treats PCOS as a lifelong condition and emphasizes menstrual irregularity, cardiometabolic assessment, psychological features, patient priorities, and shared decision-making. It recommends lipid assessment, considers the 75-g oral glucose tolerance test the most accurate glycemic test in PCOS, and permits fasting glucose or HbA1c when an OGTT cannot be performed, with lower accuracy acknowledged (52). Therefore, model inputs cannot be restricted to ultrasound morphology and hormone concentrations. They must represent the clinical factors that determine both risk and the acceptability of an intervention.

### 6.4 The action gap in dynamic PCOS management

A rising metabolic or reproductive risk estimate could plausibly prompt metabolic screening, OGTT or HbA1c follow-up, liver-risk evaluation, lifestyle intervention, or ovulation-related management. These are distinct decisions with different target populations, burdens, contraindications, and time horizons. A T3 study would need to prespecify which action is triggered, compare the rule with usual care, assess calibration and false-positive burden, incorporate pregnancy intention and shared decision-making, and evaluate net benefit and clinical outcomes.

Current PCOS AI studies have not established a dynamic threshold at which these actions should be initiated. Demonstrating that a model discriminates PCOS or identifies a worsening biomarker pattern is insufficient; the clinical question is whether acting at a defined decision time improves metabolic, reproductive, psychological, or patient-centered outcomes without disproportionate testing and treatment burden.

## 7 Nonlinear ovarian reserve decline, POI, and treatment-responsive ART

Ovarian reserve decline, premature ovarian insufficiency (POI), and ovarian response during assisted reproduction pose different temporal questions. Population aging curves describe average age-related change; serial AMH measurements attempt to estimate an unobserved individual reserve process; POI is a clinically defined boundary condition with heterogeneous causes and intermittent activity; and ART generates a treatment-response feedback process in which each intervention alters subsequent observations. These distinctions are essential because a nonlinear population curve, a repeated biomarker, and a treatment-adaptive decision problem require different models and support different clinical claims.

### 7.1 Population nonlinear decline and individual AMH uncertainty

At the population level, anti-Müllerian hormone (AMH) generally declines with reproductive aging, but the mean relationship with age is nonlinear and varies across cohorts. Some population cohorts have reported an age-dependent acceleration in the mean decline, particularly at later reproductive ages (53). This finding should not be converted into a universal turning point at 40 years for all women. A population-average curve summarizes between-person patterns and does not, by itself, identify an individual inflection point, quantify a woman-specific decline rate, or determine when a clinically meaningful reproductive event will occur.

The biological target and the recorded measurement must also be separated. Latent ovarian reserve is expected to decline overall, whereas an observed AMH value is affected by analytic error, short-term biological variation, assay-platform differences, specimen handling, hormonal exposures, and lower limits of detection (90). Monotonicity or smoothness constraints may therefore be reasonable for a latent mean process, but they should not be imposed on every measured value. Cross-organ markers require the same restraint: the retinal-age-gap study associated a fundus-image-derived marker with AMH and reproductive events, but did not repeatedly model the same women over time; it is exploratory cross-sectional or associational biomarker evidence rather than a longitudinal multimodal aging trajectory (55).

Evidence on the incremental value of repeated AMH measurements is inconsistent. In the population-based Doetinchem cohort, adding the estimated AMH decline rate improved menopause discrimination only in a limited younger-age analysis; beyond that point it did not improve prediction of menopause or early menopause, and the clinically relevant models underestimated early-menopause risk in younger women (8). In contrast, the Tehran Lipid and Glucose Study reported that adding annual AMH decline to a model containing a current AMH value improved C-statistic discrimination in its cohort (56). Differences in assay, sampling interval, age distribution, outcome definition, and statistical specification may contribute to the discordance. The incremental value of repeated AMH measurements is cohort-, assay-, age-, and model-dependent rather than universally established.

Model selection should therefore begin with the estimand rather than with a preferred neural architecture. Flexible splines or generalized additive models can estimate a population mean age curve; nonlinear mixed-effects or hierarchical Bayesian models can represent individual heterogeneity; latent-process or state-space models can separate measurement noise from an unobserved reserve state; data-driven change-point models can test for rather than assume an inflection; and joint longitudinal-event models can connect biomarker history with menopause or POI events. Individual counseling additionally requires calibrated prediction intervals and an explicit decision threshold. A Transformer should not be the default framework for AMH trajectories unless the sample size, sequence length, dimensionality, and clinical question justify it. .

### 7.2 Premature ovarian insufficiency as a boundary condition

POI should not be modeled as the normal ovarian-aging curve simply shifted to an earlier age. It is a heterogeneous clinical condition defined by loss of ovarian activity before age 40 years and may arise from genetic causes, gonadotoxic chemotherapy or radiotherapy, ovarian or pelvic surgery, autoimmune disease, or causes that remain unidentified after evaluation. These mechanisms can produce abrupt injury, accelerated depletion, impaired follicular function, or mixed patterns rather than a single smooth decline. A model trained only on usual-age AMH distributions may therefore treat clinically important POI phenotypes as tail observations instead of a distinct diagnostic boundary condition (57).

Ovarian activity in POI may also be intermittent. Menstrual bleeding, follicular activity, estradiol secretion, and FSH concentrations can fluctuate, and a temporarily normal measurement does not exclude the condition. Current guidance bases diagnosis on menstrual disturbance before age 40 years, usually amenorrhea or irregular cycles for at least four months, together with biochemical confirmation of ovarian insufficiency; an elevated FSH concentration is central, with repeat testing when diagnostic uncertainty remains. The diagnostic target is therefore a clinical-biochemical state, not a single low reserve marker or an assumed irreversible trajectory (57).

AMH has a restricted role in this setting. The current international POI guideline states that AMH should not be used as the primary diagnostic test. It may be used as an adjunct when FSH results are inconclusive, interpreted alongside age, menstrual history, hormonal treatment, and the broader clinical context. The guideline also advises against routine AMH testing to predict who will develop POI because predictive accuracy is insufficient. Consequently, low AMH alone must not be presented as diagnostic of POI, and neither a static classifier nor a longitudinal model can establish irreversible infertility or precisely determine future menopause timing (57).

Reasonable AI targets are narrower and clinically actionable: identifying patients at high risk of iatrogenic POI before gonadotoxic treatment; detecting an unexpectedly rapid decline relative to age, assay, and treatment exposure; prompting specialist evaluation when menstrual and biochemical signals become discordant; and supporting timely fertility-preservation counseling. Such systems should report uncertainty, preserve clinician review, and be evaluated for referral yield, false-positive burden, access effects, and whether counseling occurs early enough to change available options. They should not replace guideline-based diagnostic confirmation or convert probabilistic reserve estimates into categorical claims about permanent reproductive capacity.

### 7.3 OvaRePred as a trajectory-inspired endocrine-age mapping model

OvaRePred (HerTempo) was developed from a single-center retrospective cohort of GnRH-antagonist ART cycles rather than from a longitudinal community cohort. After repeated cycles from the same women were removed, the training set contained 15,241 cycles from 2017–2019 and the temporally separated test set contained 14,498 cycles from 2020-2021. The primary observed endpoint was poor ovarian response (POR), defined as retrieval of fewer than five oocytes. Thus, the empirical foundation is a large same-center ART dataset with one analyzed cycle per woman, not repeated long-term AMH measurements within individuals (36).

The core prediction model is logistic regression using age and AMH. The selected polynomial specification represents age with a quadratic term and AMH with a cubic term and predicts the probability of POR. Its discrimination was similar to simpler continuous specifications, while calibration was reported to be better in the same-center test period. This is a nonlinear static prediction task based on variables available for a current ART assessment; the model does not estimate a woman-specific AMH slope or update a latent reserve state as new longitudinal measurements arrive (36).

The endocrine-age output is produced by a second mapping step. Predicted POR probability is transformed into an ovarian-reserve score and located on a population-level two-parameter logistic curve relating age to the prevalence of predicted POR. The corresponding age on that curve is labeled endocrine age. This is population-based nonlinear mapping: it can translate a current risk estimate into a more intuitive relative position on a population curve and may provide potential support for risk communication, but it is not direct observation of an individual ovarian-aging trajectory (36).

Future reproductive milestones are then projected from the population curve under a fixed-interval hypothesis. Because OvaRePred does not contain long-term repeated AMH from the same women, does not directly estimate individual decline rates, and derives milestone timing from cross-sectional population structure, these outputs are hypothesis-generating projections. The original authors likewise call for prospective validation in longitudinal non-ART cohorts. OvaRePred should therefore be described as a trajectory-inspired T0 endocrine-age mapping model, not a T2 longitudinal aging clock, and its current role is risk communication and hypothesis generation rather than precise fertility-preservation timing (36).

### 7.4 ART as a treatment-response feedback process

Controlled ovarian stimulation is intrinsically longitudinal because treatment and observation co-evolve. A prior gonadotropin dose affects later estradiol concentrations and follicle growth; clinicians alter subsequent doses in response to those findings; and the timing of the next ultrasound or laboratory test depends on the previous response. This creates time-varying confounding and an endogenous observation process. Historical records therefore contain a mixture of ovarian biology, clinician behavior, center protocol, and patient access. A model that predicts what was prescribed can be accurate without identifying the treatment rule that would produce the best patient outcome.

Measurement automation is the first evidence layer. Automated follicle counting and measurement can improve reproducibility, reduce manual workload, and standardize the imaging variables available during stimulation (58). These systems address measurement quality. They do not, by themselves, select a gonadotropin dose, specify a trigger rule, or establish that acting on an automated measurement improves mature-oocyte yield, safety, pregnancy, or live birth. Measurement automation should therefore be evaluated separately from prescription and treatment-effect models.

Prescription prediction is the second layer. The daily FSH study analyzed 13,788 IVF/ICSI cycles retrospectively at a single center and encoded static variables together with follicle and hormone information across stimulation days. Its output was a model-recommended daily FSH dose, evaluated primarily by agreement with historical dose categories and related predictive metrics (34). Because the model explicitly used ordered within-cycle information, it is T2 time-aware prescription prediction. However, its target labels were clinicians’ prior prescriptions. Agreement with those labels does not show that the recommended dose is biologically best, reduce OHSS, improve oocyte competence, or increase live birth.

This distinction is causal, not semantic. Prior dose influences subsequent hormone and follicle response; clinicians change dose because of prior response; monitoring times depend on earlier findings; and treatment may be stopped or modified for reasons incompletely represented in the record. Historical prescriptions are behavior labels, not optimal-treatment labels. Estimating a beneficial dynamic treatment strategy requires methods that compare plausible treatment sequences under explicit assumptions about time-varying confounding, positivity, consistency, and clinically relevant outcomes. A high prescription-matching score alone cannot answer that counterfactual question.

Prospective clinician-facing decision support is the third layer. The prospective ART study (35) evaluated starting-dose and trigger tools across two centers and four physicians, with 291 treatment-arm cycles compared with matched historical controls. Clinicians could select any dose, continue stimulation, trigger, or ignore a recommendation after reviewing the model output. The platform was therefore integrated into a real decision process with human override and is an early T3 candidate. Nevertheless, the average mature-oocyte count was 12.20 versus 11.24 (p = 0.16), and total FSH use was 3671.95 versus 3846.29 IU (p = 0.13); neither primary difference reached statistical significance (35). The study was prospectively evaluated but not yet conclusively shown to improve clinical outcomes.

A conclusive T3 ART study would prespecify the action rule, record adherence and override reasons, compare the strategy with an appropriate concurrent control, and assess calibration, treatment burden, OHSS, cycle cancellation, mature oocytes, embryo outcomes, cumulative live birth, and patient-reported outcomes. It should also evaluate whether performance transports across stimulation protocols, centers, assays, and populations. Until such evidence is available, the defensible terminology is measurement automation, time-aware prescription prediction, and clinician-facing decision support—not automated dosing or a real-time closed loop.

## 8 Clinical translation and the constrained role of large language models

Clinical translation is not achieved when a model merely processes longitudinal records or improves discrimination. The model must be specified for a clinical purpose, supplied with biologically interpretable data, validated in the population and time period of intended use, linked to a defensible action threshold, and evaluated prospectively within the workflow that will act on its output. This requirement is especially important in ovarian health, where the same numerical change can have different meanings across menstrual phases, treatment stages, assays, and care pathways. The following deployment gates operationalize the transition from T0–T2 evidence toward T3 decision-linked evaluation.

### 8.1 Five deployment gates for ovarian-health AI

Before an ovarian-health AI system is described as clinically deployable, it should pass five sequential gates. These gates do not certify a model on the basis of AUROC, architectural novelty, or sequence-processing capacity alone. They reflect convergent requirements in contemporary prediction-model reporting, early live clinical evaluation, and total-product-lifecycle governance: explicit purpose, data provenance, transportable validity, human-centered decision utility, and post-deployment monitoring (93–95). Failure at an earlier gate cannot be compensated for by a more complex model at a later stage.

#### 8.1.1 Gate 1—clinical purpose

The target population, intended use, comparator, exclusion criteria, and intended action must be prespecified. A model for post-treatment ovarian-cancer recurrence is not interchangeable with a screening model for incident disease; a PCOS diagnostic classifier is not automatically a metabolic-surveillance tool; and an ART prescription model is not an autonomous dosing system. The comparator should reflect the decision currently available to clinicians, such as a fixed biomarker threshold, a validated clinical score, standard follow-up, or unaided specialist judgment. Exclusions should identify populations in whom the data-generating process or action pathway differs materially, including pregnancy, recent hormonal treatment, specific stimulation protocols, prior gonadotoxic therapy, or incomplete staging. Most importantly, the output must be tied to an intended action—for example, repeat testing, additional imaging, specialist referral, altered monitoring, or clinician review. Without a prespecified action, a model remains prognostic or time-aware evidence rather than T3 decision support (93, 94).

#### 8.1.2 Gate 2—minimum data

The minimum deployable dataset must preserve the temporal and biological context required to interpret each measurement. Exact timestamps are necessary but insufficient; records should also identify menstrual-cycle phase, ovulatory status when available, treatment or stimulation stage, surgery and chemotherapy dates, recurrence-related events, medication exposure, and the sequence of prior actions. Core clinical variables should be defined before modeling rather than selected only because they are conveniently recorded. Assay platforms, units, detection limits, reference intervals, imaging protocols, and segmentation or measurement procedures require harmonization or explicit modeling because apparent temporal change may otherwise reflect a laboratory or imaging transition rather than biological progression. The minimum dataset should also document why and by whom a test was ordered, because observation times may be clinician-driven. A model should not be deployed when required phase or treatment-state variables are systematically unavailable, even if missingness indicators improve internal prediction (93, 95).

#### 8.1.3 Gate 3—validity

Internal cross-validation is insufficient for deployment. Evaluation should include external validation across centers or health systems and temporal validation in a later period, with discrimination accompanied by calibration-in-the-large, calibration slope, clinically relevant calibration plots, and uncertainty estimates. Performance should be examined across age and reproductive stage, disease subtype, race and ethnicity where recorded appropriately, socioeconomic and geographic groups, assay and imaging platforms, and treatment protocols. For time-updated models, validation must reproduce the intended update schedule and prevent leakage from future measurements or actions. Uncertainty should influence presentation and escalation: a wide prediction interval, out-of-distribution input, or missing phase information should trigger abstention or human review rather than a falsely precise risk estimate. Subgroup analysis should be planned around plausible transportability failures rather than used only after an overall result appears favorable. A model that transports poorly or is mis calibrated in the intended population has not crossed this gate, regardless of its development-set performance (93, 96).

#### 8.1.4 Gate 4—decision utility

A validated risk estimate becomes clinically relevant only when linked to an action threshold whose benefits and harms are evaluated. The threshold should specify what happens above and below it, the acceptable false-positive and false-negative burden, the frequency of repeat testing, and whether the action is reversible. Decision-curve or related net-benefit analyses should use clinically credible threshold ranges and should not substitute for prospective evaluation when the action can cause treatment toxicity, anxiety, cost, or loss of reproductive opportunity. Clinician override must be available, recorded, and analyzed; systematic overrides may reveal unmodeled contraindications, patient preferences, workflow barriers, or model error. The MRC OV05/EORTC 55955 trial illustrates the central distinction: earlier detection of a CA-125 rise did not translate into improved overall survival when it triggered earlier chemotherapy (9). Therefore, improved temporal discrimination is not itself evidence that an AI-triggered action is beneficial (94).

#### 8.1.5 Gate 5—prospective implementation

Deployment should proceed in stages. A silent-deployment phase can test data availability, timing, calibration, and alert volume without exposing clinicians or patients to recommendations. A workflow pilot should then evaluate usability, latency, alert routing, failure handling, clinician comprehension, override reasons, and whether the model changes decisions as intended. An impact study with an appropriate concurrent comparator should assess patient-relevant outcomes, process measures, safety events, resource use, and unintended consequences rather than only agreement with historical decisions. After implementation, monitoring should detect changes in population mix, assays, imaging equipment, documentation, treatment protocols, and outcome prevalence. Prespecified procedures are needed for drift investigation, recalibration, version control, rollback, and communication of model changes. A system should be suspended when required inputs are unavailable or performance falls outside predefined limits. Only a model that remains calibrated, supports a defined action, preserves human override, and demonstrates acceptable prospective impact can be considered mature T3 evidence (94, 95).

### 8.2 LLMs as an interface rather than a trajectory engine

Large language models can assist with language-intensive tasks, but their ability to process a chronological narrative does not make them estimators of latent ovarian state, recurrence hazard, or treatment effect. A defensible role is to extract menstrual, laboratory, imaging, and treatment timelines from text; identify missing phase or treatment context; retrieve relevant guideline passages; explain outputs from separately validated numerical models; generate patient-facing summaries; and document shared decisions. These functions should use structured templates, source-linked retrieval, explicit uncertainty, and task-specific output constraints. Numerical risk estimation, dose selection, and action thresholds should remain within validated models and clinical protocols rather than being inferred from unconstrained text generation.

LLMs should serve as constrained interfaces connecting validated numerical models, clinical guidelines, and human decision-makers rather than as autonomous ovarian trajectory or treatment engines. They should not silently alter a risk score, replace missing biological context with plausible text, or convert a descriptive association into a treatment recommendation. A clinician-facing interface should display the source data and timestamps used, identify information that could not be verified, preserve the original numerical output and its calibration context, and require confirmation before any recommendation enters the medical record. For patient communication, the system should distinguish ovarian reserve from natural fecundability, population risk from individual certainty, and decision support from a diagnosis or prognosis. Human review is required whenever the output could influence chemotherapy, fertility preservation, gonadotropin dosing, or the interpretation of POI.

### 8.3 Ovarian-specific failure modes and human oversight

Liu et al. (97) evaluated the quality of LLM-generated ART chains of clinical reasoning from standardized cases; it was a synthetic reasoning-data evaluation, not a patient-management system or an autonomous decision trial. Selective few-shot prompting with diverse, high-quality examples improved senior-expert ratings of logical clarity, use of key information, and clinical accuracy, whereas random few-shot prompting did not improve on zero-shot prompting. The GPT-4o evaluator did not reliably distinguish the performance differences detected by clinicians, showing that automated scoring cannot replace expert assessment of reproductive-medicine reasoning (97).

Ovarian-specific errors require explicit safeguards. An LLM may ignore menstrual-cycle phase; conflate diminished ovarian reserve, POI, and normal reproductive aging; interpret low AMH as proof that natural conception is impossible; treat historical physician prescriptions as optimal doses; recommend chemotherapy directly from a CA-125 change; omit the patient’s pregnancy intention; or ignore assay platform, units, and reference intervals. These errors can appear linguistically coherent while violating the evidence boundaries established elsewhere in this review (8, 9, 34, 52, 57). Human oversight should therefore use task-specific checklists and hard stops: verify phase and treatment state, display the original laboratory context, distinguish diagnosis from risk communication, prohibit direct generation of medication or chemotherapy orders, and document the clinician’s acceptance, modification, or rejection of any suggested text.

General representation bias remains relevant but should not displace ovarian-specific validation. Training data concentrated in particular countries, centers, racial or socioeconomic groups, or patterns of healthcare use can produce unequal errors, and LLM-generated recommendations may reproduce demographic stereotypes or stigmatizing language. Fairness evaluation should therefore examine both numerical performance and the content of generated explanations across clinically relevant subgroups, while recognizing that visit frequency, cost, and record completeness may act as proxies for access rather than biological need. These risks require representative data, subgroup auditing, source verification, and accountable human review throughout development and deployment (80, 81, 98–102).

### 8.4 Limitations of this review

This review has limitations. It was designed as a structured narrative review rather than an exhaustive systematic review or meta-analysis. Representative primary studies were selected for design-level verification because they supported major claims or were frequently characterized as longitudinal, dynamic, trajectory-based, or clinically actionable. Although two authors independently verified these studies, study selection and the author-defined T0–T3 classification remain subject to judgment. No formal risk-of-bias tool was applied across all primary studies, and heterogeneous populations, outcomes, and analytical tasks precluded quantitative pooling. The T0–T3 framework has not undergone external validation or formal inter-rater reliability testing beyond the consensus classification used in this review. It should therefore be interpreted as an analytical framework for temporal-information use and decision linkage, not as an externally validated quality or maturity scale.

## 9 Conclusions and future perspectives

Five conclusions emerge from this cross-context analysis. First, Longitudinality depends on how within-patient time is modeled, not merely on repeated data collection. Repeated observations may still constitute T1 evidence when temporal information is compressed into velocity, slope, half-life, nadir, change score, or area under the curve. T2 requires explicit use of measurement order, elapsed intervals, time-varying covariates, missingness patterns, latent states, or risk updating as new data arrive, whereas T3 additionally connects the output to a prespecified clinical action and evaluates calibration, false-positive burden, net benefit, clinician override, and prospective impact. T0–T3 therefore describe how temporal information is used and linked to clinical decisions; they do not represent an automatic hierarchy of model quality.

Second, Current literature frequently conflates ovarian longitudinal biology, direct ovarian AI, and transferred methodology. Long-term PCOS cohorts and repeated-AMH studies define biological processes that a future model may need to represent; static pathology and radiomics studies establish prognostic associations at a single time point; and serial imaging work in lung cancer illustrates a potentially transferable sequence-modeling strategy. These forms of evidence are all relevant, but they are not interchangeable and should not be presented as direct validation of longitudinal ovarian AI (8, 20, 37, 39–47, 49–51, 53).

Third, Ovarian clinical contexts have different biological-time structures and cannot be served by one universal architecture. PCOS is phase-dependent, state-switching, and multiscale; ovarian reserve decline is a latent nonlinear process observed through noisy and assay-dependent biomarkers; POI is a heterogeneous boundary condition that is not simply normal aging shifted earlier; ART is a treatment–response feedback system in which previous doses alter later measurements and clinician decisions; and ovarian malignancy combines event risk, treatment perturbation, and symptom- or biomarker-triggered observation. Model selection should therefore follow the clinical estimand and observation process rather than the availability of a fashionable architecture. A Transformer is not a longitudinal model merely because it can process sequences.

Fourth, Direct evidence remains concentrated in T0–T2; convincing T3 impact evidence is limited. Among the review-level evidence mapped and representative primary studies verified, PCOS diagnostic models, ovarian-cancer pathology and imaging studies, and ovarian-reserve tools were predominantly T0. CA-125 velocity, half-life, and nadir are principally T1 serial-feature evidence. The daily FSH model is a T2 time-aware prescription-prediction study because it uses ordered stimulation data, but it was trained retrospectively to reproduce historical prescriptions rather than to identify a biologically optimal treatment policy (31–34, 37, 39–46). The prospective ART decision-support study is an early T3 candidate because clinicians could accept or reject recommendations, yet differences in mature oocytes and total FSH were not statistically significant (35). Improved discrimination, earlier warning, or closer agreement with clinician behavior should not be equated with clinical benefit.

Fifth, LLMs are more defensible as constrained interfaces than as autonomous predictors or treatment engines. Their appropriate roles include extracting menstrual and treatment timelines, identifying missing context, retrieving guidelines, explaining outputs from validated numerical models, generating patient-facing summaries, and documenting shared decisions. Liu et al. (97) evaluated the quality of synthetic ART reasoning chains rather than a patient-management system; selective few-shot prompting improved expert ratings, but the GPT-4o evaluator did not reliably distinguish clinically meaningful differences between prompting strategies. Human gold standards, restricted task definitions, source traceability, and explicit escalation rules are therefore required before LLMs enter ovarian-health workflows (97).

Future work should address these limitations directly. The scarcity of direct ovarian longitudinal AI requires multicenter cohorts with repeated measurements from the same women, rather than additional cross-sectional inventories. Biological-time misalignment requires standardized recording of menstrual phase, ovulatory status, treatment stage, event time, medication exposure, and exact timestamps, together with assay and imaging harmonization. Because repeated-AMH results differ across cohorts, future reserve models should separate latent decline from measurement error, quantify prediction intervals, and undergo age-, assay-, and population-specific external calibration; the conflicting Doetinchem and Tehran findings should be treated as a design constraint rather than resolved by a universal claim (8, 56). OvaRePred should be evaluated prospectively in non-ART community cohorts before population-curve projections are used for individualized reproductive-milestone counseling (36).

ART research must move from learning historical clinician behavior to estimating treatment effects. This requires causal longitudinal analyses that account for time-varying confounding, followed by outcome-oriented trials evaluating safety, mature oocytes, cumulative live birth, medication burden, patient experience, and clinician override rather than prescription agreement alone (34, 35). In ovarian cancer, the central question is not whether CA-125 or an updated risk score can identify recurrence earlier, but which threshold should trigger repeat biomarker testing, imaging, shortened follow-up, trial referral, or treatment in a subgroup with positive net benefit. The lack of an observed overall-survival benefit from CA-125-triggered early treatment demonstrates why temporal discrimination and action utility must be evaluated separately (9). Static pathology, CT, PET/CT, and MRI studies should no longer be labelled longitudinal; direct progress requires true multi-time-point ovarian imaging with explicit interval and treatment modeling (37, 39–47).

Finally, translation studies should use staged implementation: silent deployment, workflow pilots, prospective impact evaluation, drift monitoring, and recalibration. LLM-generated outputs and automated evaluators should be compared with blinded human expert standards within restricted workflows, with audit trails for acceptance, modification, and override (93–95, 97). Across domains, success should be judged against an explicit clinical comparator: usual surveillance in ovarian cancer, guideline-based metabolic follow-up in PCOS, calibrated counseling in ovarian aging and POI, and clinician-led stimulation in ART. Reports should make the temporal unit, update schedule, action pathway, override process, and patient-relevant outcome auditable. Without these elements, a higher AUROC or more complex sequence processor remains a technical result rather than evidence of improved care. The clinical value of temporal AI in ovarian health depends less on architectural complexity than on correct alignment with ovarian biology, direct within-patient temporal evidence, calibrated validation, and a beneficial clinical action.
