# Supplementary material for: From static snapshots to longitudinal trajectories: artificial intelligence in women’s reproductive and ovarian health
Source: Front Endocrinol (Lausanne). 2026 Aug 12;17:1893963. doi: 10.3389/fendo.2026.1893963 (PMC13507990; doi:10.3389/fendo.2026.1893963)
Supplement: Supplementary file 1 [file DataSheet1.docx]

Supplementary Methods S1. Search strategy

**From Static Snapshots to Longitudinal Trajectories: Artificial Intelligence in Women’s Reproductive and Ovarian Health**

# Scope and date limits

**Review design.** Structured narrative review with review-level evidence mapping and design-level verification of representative primary studies; the search was not presented as an exhaustive systematic review or meta-analysis.

**Eligible publication window.** 1 January 1989 through 1 January 2026, inclusive. Online-ahead-of-print articles and publicly available preprints within this window were eligible. If an eligible preprint subsequently appeared as a peer-reviewed article, the version of record was cited without changing the evidence set.

**Databases and supplementary sources.** PubMed/MEDLINE and Web of Science Core Collection were searched using two complementary streams. Targeted checks of arXiv and online-ahead-of-print sections of relevant medical and computational journals were used to identify eligible preprints and updated versions of record.

# Search stream 1: temporal-evidence search

Purpose: identify ovarian-health studies using repeated measurements, explicit time representations, dynamic prediction, or relevant longitudinal statistical models.

## PubMed/MEDLINE—temporal-evidence stream

(("Ovarian Reserve"[Mesh] OR "Anti-Mullerian Hormone"[Mesh] OR "Polycystic Ovary Syndrome"[Mesh] OR "Ovarian Neoplasms"[Mesh] OR "Cystadenocarcinoma, Serous"[Mesh] OR "Reproductive Techniques, Assisted"[Mesh] OR "Menopause"[Mesh] OR "Primary Ovarian Insufficiency"[Mesh] OR "ovarian reserve"[tiab] OR "anti-Mullerian hormone"[tiab] OR "anti-Müllerian hormone"[tiab] OR AMH[tiab] OR "polycystic ovary syndrome"[tiab] OR PCOS[tiab] OR "ovarian cancer"[tiab] OR "ovarian neoplasm*"[tiab] OR "ovarian malignan*"[tiab] OR "high-grade serous ovarian carcinoma"[tiab] OR HGSOC[tiab] OR "ovarian aging"[tiab] OR "ovarian ageing"[tiab] OR "ovarian function"[tiab] OR "premature ovarian insufficiency"[tiab] OR "primary ovarian insufficiency"[tiab] OR "premature ovarian failure"[tiab] OR POI[tiab] OR "assisted reproductive technolog*"[tiab] OR "in vitro fertilization"[tiab] OR "in vitro fertilisation"[tiab] OR IVF[tiab] OR ICSI[tiab] OR "controlled ovarian stimulation"[tiab]) AND ("Longitudinal Studies"[Mesh] OR "Cohort Studies"[Mesh] OR "Electronic Health Records"[Mesh] OR "Survival Analysis"[Mesh] OR longitudinal[tiab] OR cohort[tiab] OR "time series"[tiab] OR temporal[tiab] OR dynamic[tiab] OR "repeated measure*"[tiab] OR "serial measure*"[tiab] OR "electronic health record*"[tiab] OR "electronic medical record*"[tiab] OR EHR[tiab] OR EMR[tiab] OR "follow-up"[tiab] OR "follow up"[tiab] OR prognos*[tiab] OR predict*[tiab] OR recurrence[tiab] OR survival[tiab] OR "joint model*"[tiab] OR landmark*[tiab] OR "state space"[tiab] OR "state-space"[tiab] OR "mixed effect*"[tiab] OR "latent process*"[tiab] OR "functional data analysis"[tiab] OR "missing data"[tiab] OR missingness[tiab] OR "time-varying"[tiab] OR "time varying"[tiab]) AND (("Artificial Intelligence"[Mesh] OR "Machine Learning"[Mesh] OR "Deep Learning"[Mesh] OR "Neural Networks, Computer"[Mesh] OR "Natural Language Processing"[Mesh] OR "Large Language Models"[Mesh] OR "artificial intelligence"[tiab] OR AI[tiab] OR "machine learning"[tiab] OR "deep learning"[tiab] OR "neural network*"[tiab] OR "representation learning"[tiab] OR "long short-term memory"[tiab] OR LSTM[tiab] OR LSTMs[tiab] OR Transformer*[tiab] OR attention[tiab] OR "large language model*"[tiab] OR LLM[tiab] OR LLMs[tiab] OR "foundation model*"[tiab] OR multimodal[tiab] OR radiomic*[tiab]) OR ("Models, Statistical"[Mesh] OR "joint model*"[tiab] OR landmark*[tiab] OR "state space model*"[tiab] OR "state-space model*"[tiab] OR "mixed-effects model*"[tiab] OR "mixed effects model*"[tiab] OR "hierarchical Bayesian"[tiab] OR "latent-process model*"[tiab] OR "latent process model*"[tiab] OR "functional data analysis"[tiab] OR "dynamic prediction"[tiab]))) AND ("1989/01/01"[dp] : "2026/01/01"[dp])

## Web of Science Core Collection—temporal-evidence stream

TS=("ovarian reserve" OR "anti-Mullerian hormone" OR "anti-Müllerian hormone" OR AMH OR "polycystic ovary syndrome" OR PCOS OR "ovarian cancer" OR "ovarian neoplasm*" OR "ovarian malignan*" OR "high-grade serous ovarian carcinoma" OR HGSOC OR "ovarian aging" OR "ovarian ageing" OR "ovarian function" OR "premature ovarian insufficiency" OR "primary ovarian insufficiency" OR "premature ovarian failure" OR POI OR "assisted reproductive technolog*" OR "in vitro fertilization" OR "in vitro fertilisation" OR IVF OR ICSI OR "controlled ovarian stimulation") AND TS=(longitudinal OR cohort OR "time series" OR temporal OR dynamic OR "repeated measure*" OR "serial measure*" OR "electronic health record*" OR "electronic medical record*" OR EHR OR EMR OR "follow-up" OR "follow up" OR prognos* OR predict* OR recurrence OR survival OR "joint model*" OR landmark* OR "state space" OR "state-space" OR "mixed effect*" OR "latent process*" OR "functional data analysis" OR "missing data" OR missingness OR "time-varying" OR "time varying") AND (TS=("artificial intelligence" OR AI OR "machine learning" OR "deep learning" OR "neural network*" OR "representation learning" OR "long short-term memory" OR LSTM OR LSTMs OR Transformer* OR attention OR "large language model*" OR LLM OR LLMs OR "foundation model*" OR multimodal OR radiomic*) OR TS=("joint model*" OR landmark* OR "state space model*" OR "state-space model*" OR "mixed-effects model*" OR "mixed effects model*" OR "hierarchical Bayesian" OR "latent-process model*" OR "latent process model*" OR "functional data analysis" OR "dynamic prediction")) AND DOP=1989-01-01/2026-01-01

# Search stream 2: domain-specific AI search

Purpose: identify static or cross-sectional ovarian-health AI studies and prior reviews that could be missed by requiring temporal terminology.

## PubMed/MEDLINE—domain-specific AI stream

(("Ovarian Reserve"[Mesh] OR "Anti-Mullerian Hormone"[Mesh] OR "Polycystic Ovary Syndrome"[Mesh] OR "Ovarian Neoplasms"[Mesh] OR "Cystadenocarcinoma, Serous"[Mesh] OR "Reproductive Techniques, Assisted"[Mesh] OR "Menopause"[Mesh] OR "Primary Ovarian Insufficiency"[Mesh] OR "ovarian reserve"[tiab] OR "anti-Mullerian hormone"[tiab] OR "anti-Müllerian hormone"[tiab] OR AMH[tiab] OR "polycystic ovary syndrome"[tiab] OR PCOS[tiab] OR "ovarian cancer"[tiab] OR "ovarian neoplasm*"[tiab] OR "ovarian malignan*"[tiab] OR "high-grade serous ovarian carcinoma"[tiab] OR HGSOC[tiab] OR "ovarian aging"[tiab] OR "ovarian ageing"[tiab] OR "ovarian function"[tiab] OR "premature ovarian insufficiency"[tiab] OR "primary ovarian insufficiency"[tiab] OR "premature ovarian failure"[tiab] OR POI[tiab] OR "assisted reproductive technolog*"[tiab] OR "in vitro fertilization"[tiab] OR "in vitro fertilisation"[tiab] OR IVF[tiab] OR ICSI[tiab] OR "controlled ovarian stimulation"[tiab]) AND ("Artificial Intelligence"[Mesh] OR "Machine Learning"[Mesh] OR "Deep Learning"[Mesh] OR "Neural Networks, Computer"[Mesh] OR "Natural Language Processing"[Mesh] OR "Large Language Models"[Mesh] OR "artificial intelligence"[tiab] OR AI[tiab] OR "machine learning"[tiab] OR "deep learning"[tiab] OR "neural network*"[tiab] OR "representation learning"[tiab] OR "long short-term memory"[tiab] OR LSTM[tiab] OR LSTMs[tiab] OR Transformer*[tiab] OR attention[tiab] OR "large language model*"[tiab] OR LLM[tiab] OR LLMs[tiab] OR "foundation model*"[tiab] OR multimodal[tiab] OR radiomic*[tiab])) AND ("1989/01/01"[dp] : "2026/01/01"[dp])

## Web of Science Core Collection—domain-specific AI stream

TS=("ovarian reserve" OR "anti-Mullerian hormone" OR "anti-Müllerian hormone" OR AMH OR "polycystic ovary syndrome" OR PCOS OR "ovarian cancer" OR "ovarian neoplasm*" OR "ovarian malignan*" OR "high-grade serous ovarian carcinoma" OR HGSOC OR "ovarian aging" OR "ovarian ageing" OR "ovarian function" OR "premature ovarian insufficiency" OR "primary ovarian insufficiency" OR "premature ovarian failure" OR POI OR "assisted reproductive technolog*" OR "in vitro fertilization" OR "in vitro fertilisation" OR IVF OR ICSI OR "controlled ovarian stimulation") AND TS=("artificial intelligence" OR AI OR "machine learning" OR "deep learning" OR "neural network*" OR "representation learning" OR "long short-term memory" OR LSTM OR LSTMs OR Transformer* OR attention OR "large language model*" OR LLM OR LLMs OR "foundation model*" OR multimodal OR radiomic*) AND DOP=1989-01-01/2026-01-01

# Review-level evidence mapping

Separate review-level searches were conducted for six domains. The exact domain blocks below were combined with the AI and review filters shown after the table, plus the same publication-date restriction.

| **Review domain** | **PubMed domain block** | **Web of Science domain block** |
| --- | --- | --- |
| **Ovarian cancer AI reviews** | ("Ovarian Neoplasms"[Mesh] OR "ovarian cancer"[tiab] OR "ovarian neoplasm*"[tiab] OR "ovarian malignan*"[tiab] OR HGSOC[tiab]) | TS=("ovarian cancer" OR "ovarian neoplasm*" OR "ovarian malignan*" OR HGSOC) |
| **PCOS AI reviews** | ("Polycystic Ovary Syndrome"[Mesh] OR "polycystic ovary syndrome"[tiab] OR PCOS[tiab]) | TS=("polycystic ovary syndrome" OR PCOS) |
| **Ovarian reserve/aging/POI AI reviews** | ("Ovarian Reserve"[Mesh] OR "Anti-Mullerian Hormone"[Mesh] OR "Primary Ovarian Insufficiency"[Mesh] OR "ovarian reserve"[tiab] OR AMH[tiab] OR "ovarian aging"[tiab] OR "ovarian ageing"[tiab] OR "premature ovarian insufficiency"[tiab] OR "primary ovarian insufficiency"[tiab] OR POI[tiab]) | TS=("ovarian reserve" OR AMH OR "ovarian aging" OR "ovarian ageing" OR "premature ovarian insufficiency" OR "primary ovarian insufficiency" OR POI) |
| **ART AI reviews** | ("Reproductive Techniques, Assisted"[Mesh] OR "assisted reproductive technolog*"[tiab] OR "in vitro fertilization"[tiab] OR "in vitro fertilisation"[tiab] OR IVF[tiab] OR ICSI[tiab] OR "controlled ovarian stimulation"[tiab]) | TS=("assisted reproductive technolog*" OR "in vitro fertilization" OR "in vitro fertilisation" OR IVF OR ICSI OR "controlled ovarian stimulation") |
| **Reproductive medicine LLM reviews** | (("Infertility"[Mesh] OR infertility[tiab] OR "reproductive medicine"[tiab] OR "assisted reproduction"[tiab] OR IVF[tiab]) AND ("Large Language Models"[Mesh] OR "large language model*"[tiab] OR LLM[tiab] OR LLMs[tiab] OR ChatGPT[tiab])) | TS=((infertility OR "reproductive medicine" OR "assisted reproduction" OR IVF) AND ("large language model*" OR LLM OR LLMs OR ChatGPT)) |
| **Longitudinal clinical AI reviews** | (("Electronic Health Records"[Mesh] OR "electronic health record*"[tiab] OR EHR[tiab] OR longitudinal[tiab] OR "time series"[tiab] OR temporal[tiab]) AND ("Artificial Intelligence"[Mesh] OR "Machine Learning"[Mesh] OR "Deep Learning"[Mesh] OR "artificial intelligence"[tiab] OR "machine learning"[tiab] OR "deep learning"[tiab] OR Transformer*[tiab] OR LSTM[tiab])) | TS=(("electronic health record*" OR EHR OR longitudinal OR "time series" OR temporal) AND ("artificial intelligence" OR "machine learning" OR "deep learning" OR Transformer* OR LSTM)) |

## PubMed review filter and combination rule

For each PubMed domain block: (DOMAIN BLOCK AND ("Artificial Intelligence"[Mesh] OR "Machine Learning"[Mesh] OR "Deep Learning"[Mesh] OR "Neural Networks, Computer"[Mesh] OR "Natural Language Processing"[Mesh] OR "Large Language Models"[Mesh] OR "artificial intelligence"[tiab] OR AI[tiab] OR "machine learning"[tiab] OR "deep learning"[tiab] OR "neural network*"[tiab] OR "representation learning"[tiab] OR "long short-term memory"[tiab] OR LSTM[tiab] OR LSTMs[tiab] OR Transformer*[tiab] OR attention[tiab] OR "large language model*"[tiab] OR LLM[tiab] OR LLMs[tiab] OR "foundation model*"[tiab] OR multimodal[tiab] OR radiomic*[tiab]) AND ("Review"[Publication Type] OR "Meta-Analysis"[Publication Type] OR review[tiab] OR "systematic review"[tiab] OR "scoping review"[tiab] OR "narrative review"[tiab] OR meta-analy*[tiab])) AND ("1989/01/01"[dp] : "2026/01/01"[dp])

## Web of Science review filter and combination rule

For each Web of Science domain block: DOMAIN BLOCK AND TS=("artificial intelligence" OR AI OR "machine learning" OR "deep learning" OR "neural network*" OR "representation learning" OR "long short-term memory" OR LSTM OR LSTMs OR Transformer* OR attention OR "large language model*" OR LLM OR LLMs OR "foundation model*" OR multimodal OR radiomic*) AND TS=(review OR "systematic review" OR "scoping review" OR "narrative review" OR meta-analy*) AND DOP=1989-01-01/2026-01-01

# Targeted preprint and online-ahead-of-print checks

arXiv was checked using combinations of ovarian-health terms (ovarian cancer, PCOS, ovarian reserve, ovarian aging, POI, ART, IVF) with artificial intelligence, machine learning, longitudinal, time series, temporal, multimodal, and large language model terms. Candidate records were screened by title and abstract for relevance to the four ovarian-health contexts.

Online-ahead-of-print sections of journals identified during database searching were checked for eligible articles dated on or before 1 January 2026.

When a preprint eligible by the cutoff later had a peer-reviewed version of record, the peer-reviewed citation replaced the preprint citation while the eligibility date and evidence set remained unchanged.

# Study identification and design-level verification

Review-level records were used to map prior coverage, identify duplicated claims, and locate representative primary studies. Review conclusions were not treated as proof that time-aware AI had been implemented in an ovarian-health population.

Representative primary studies supporting major claims were checked in full text for population, study design, repeated within-patient measurements, actual temporal representation, outcome, comparator, validation, and clinical action.

Two authors independently performed the design-level verification and T0-T3 classification; disagreements were resolved by consensus. Classification followed the actual data structure and analytical procedure rather than terms such as “dynamic,” “longitudinal,” or “trajectory” in the title or discussion.

Studies using repeated measurements only to derive velocity, slope, half-life, nadir, change score, or area under the curve were classified as T1 serial-feature summarization. T2 required explicit use of order, intervals, time-varying covariates, missingness, latent states, or risk updating. T3 required linkage to a prespecified clinical action and evaluation of decision-relevant consequences.

**Supplementary Table S1. Comparison of previous reviews and the analytical contribution of the present review**

| **Domain** | **Study scope** | **Longitudinal AI** | **Multimodal fusion** | **Ovarian biological time** | **Evidence provenance** | **Clinical inputs** | **Action thresholds** | **Present-review difference** |
| --- | --- | --- | --- | --- | --- | --- | --- | --- |
| Ovarian cancer histopathology | Breen et al., 2023 (Supplementary Ref. S1); systematic review of 45 studies and 80 histopathology models. | No formal within-patient temporal analysis; models were predominantly single-slide T0 analyses. | Limited; mainly histopathology, with occasional clinicopathological covariates. | Not addressed. | Risk of bias and reporting were assessed, but evidence was not separated into direct ovarian AI, ovarian longitudinal biology, and transferred methodology. | Whole-slide images and selected clinicopathological variables. | No temporal action rule or net-benefit assessment. | Reclassifies pathology as static T0 unless same-patient order, intervals, and risk updating are explicitly modeled. |
| Ovarian cancer ultrasound | Mitchell et al., 2024 (Supplementary Ref. S2); systematic review and meta-analysis of 14 ultrasound-only diagnostic studies. | No; inputs were generally single-encounter ultrasound images. | Low by design; ultrasound image information was the principal input. | Not addressed. | Diagnostic evidence was summarized without a temporal-evidence provenance framework. | 2D/3D ultrasound images; no longitudinal treatment history. | Diagnostic cutoffs were assessed, not longitudinal clinical-action thresholds. | Separates static adnexal-mass diagnosis from recurrence monitoring and decision-linked temporal assessment. |
| Gynecologic malignancies | Zhang and Qin, 2025 (main-text Ref. 19); narrative review of deep learning across gynecologic malignancies. | RNN/Transformer concepts were discussed, but ovarian patient-level temporal designs were not systematically verified. | Yes; pathology, imaging, omics, and clinical data. | Not explicitly analyzed. | No formal provenance categories. | Imaging, pathology, omics, EHR, and clinical variables. | Limited evaluation of calibrated action thresholds or net benefit. | Uses design-level verification and T0-T3 classification rather than inferring longitudinality from architecture. |
| PCOS | Barrera et al., 2023 (main-text Ref. 20); systematic review of 31 diagnosis/classification studies. | No substantive patient-level risk updating; evidence was mainly T0 diagnosis/classification. | Partial; clinical data with or without imaging, plus EHR, genetic, and proteomic data. | Not analyzed as phase-dependent, state-switching, or multiscale biology. | Review provenance was reported, but longitudinal biological studies were not separated from direct AI. | Clinical variables, EHR, ultrasound, genetic, and proteomic data. | Diagnostic thresholds/performance only; no dynamic action threshold. | Distinguishes static diagnosis from PCOS longitudinal biology and from future T2/T3 management models. |
| PCOS | Ghaderzadeh et al., 2025 (Supplementary Ref. S3); systematic review of 80 diagnostic, predictive, biomarker, and decision-support studies. | Temporal claims were summarized, but explicit within-person temporal representation was not the organizing criterion. | Yes; imaging, clinical/EHR, biochemical, omics, and emerging generative-AI applications. | Limited. | No formal three-source provenance framework. | Phenotype, hormones, metabolic variables, ultrasound, text, and omics. | Prespecified longitudinal action thresholds and net benefit were not consistently evaluated. | Adds phase, life-stage, medication, pregnancy-intention, exact-time, and action-linkage requirements. |
| Ovarian reserve/aging | Moustakli et al., 2025 (main-text Ref. 21); narrative review linking AI with reproductive aging biology. | Conceptual discussion of aging trajectories; direct time-aware ovarian AI remained limited. | Yes; biomarkers, omics, imaging, and biological-aging signals. | Partly discussed, but observation time and latent reserve state were not formally separated. | Biological and AI evidence were discussed together. | AMH, age, molecular aging markers, imaging, and omics. | No validated counseling or intervention threshold framework. | Separates population biology, measurement uncertainty, true within-woman modeling, and trajectory-inspired T0 mapping. |
| ART | Wu et al., 2025 (main-text Ref. 22); systematic review of 48 ART AI articles. | Partial; some cycle-aware applications, but most evidence was not classified by actual temporal input. | Yes; clinical, laboratory, imaging, gamete, and embryo data. | Treatment stage was considered, but biological stage and calendar day were not formally distinguished. | No direct/biological/transferred evidence separation. | Reserve markers, stimulation data, gamete/embryo data, and laboratory images. | Action thresholds, override, calibration, and net benefit were not consistent review axes. | Separates measurement automation, T2 prescription prediction, and early T3 decision support; treats prescriptions as behavior labels. |
| ART | Kim, 2025 (Supplementary Ref. S4); narrative review of AI across counseling, stimulation, follicular monitoring, gamete/embryo assessment, and outcome prediction. | Partial; cycle workflows were discussed without formal T0-T3 classification. | Yes. | Partly implicit through stimulation stage and prior-cycle information. | No formal evidence-provenance separation. | Age, AMH, AFC, hormones, follicle images, gamete/embryo data, and prior cycles. | Clinical utility was generally inferred from predictive performance. | Requires treatment-response feedback, time-varying confounding, clinician override, and prospective patient-relevant outcomes. |
| ART | Kakkar et al., 2025 (Supplementary Ref. S5); comprehensive review of AI in assisted reproduction, benefits, challenges, and ethics. | Temporal and adaptive claims were broad rather than design-verified. | Yes. | Limited explicit treatment of stage misalignment. | No formal provenance framework. | Stimulation, gamete/embryo images, and clinical/laboratory variables. | No systematic extraction of thresholds, false-positive burden, or net benefit. | Adds cross-context temporal evidence classification and separates prediction from beneficial action. |
| Reproductive medicine LLMs | Li et al., 2025 (Supplementary Ref. S6); systematic review of 13 infertility/sterility LLM studies. | No validated ovarian trajectory engine; studies focused on information, examinations, counseling, or question answering. | Limited; primarily text-based. | Generally absent. | LLM evaluation was not separated from numerical temporal-model evidence. | Prompts, clinical questions, educational content, and counseling responses. | No validated treatment-action thresholds. | Positions LLMs as constrained interfaces, not autonomous ovarian trajectory or treatment engines. |
| Multimodal clinical AI | Huang et al., 2020 (main-text Ref. 15); systematic review of 17 studies fusing medical imaging and EHR data. | Not the main focus; many inputs were cross-sectional. | Yes; core review topic. | Not ovarian-specific. | General methodological evidence without ovarian provenance distinctions. | Medical images plus structured or unstructured EHR variables. | No consistent decision-threshold or net-benefit evaluation. | Uses this literature only as transferred methodology unless direct ovarian temporal validation exists. |
| Longitudinal EHR AI | Carrasco-Ribelles et al., 2023 (Supplementary Ref. S7); systematic methodological review of 81 longitudinal-EHR prediction studies. | Yes; inclusion required longitudinal EHR data and a method able to use it. | Limited; imaging was not central. | Not ovarian-specific; recorded EHR time predominated. | General methodological evidence. | Diagnoses, drugs, laboratory results, procedures, and clinical measures. | Implementation and action linkage were inconsistently reported; reporting quality and risk of bias were major concerns. | Transfers methods cautiously and requires ovarian biological alignment, external/temporal validation, calibration, and action linkage. |
| EHR trajectories | Amirahmadi et al., 2023 (Supplementary Ref. S8); systematic review of 63 deep-learning EHR-trajectory studies. | Yes; focused on sequence representation and future-risk prediction. | Limited. | Not ovarian-specific. | General methodological evidence. | High-dimensional EHR events, codes, laboratory data, and treatments. | Action utility was not a primary organizing framework. | States that sequence-capable architecture alone is insufficient; direct within-patient ovarian evidence must be demonstrated. |
| Longitudinal EHR prevention | Swinckels et al., 2024 (main-text Ref. 18); scoping review of longitudinal-EHR AI for early detection and prevention. | Yes. | Limited; imaging was outside the central scope. | Not ovarian-specific. | General clinical-AI evidence. | Longitudinal primary-care and EHR variables. | Clinical outcomes and implementation evidence were sparse. | Adds ovarian evidence provenance and requires beneficial clinical action for mature T3 claims. |
| Time-varying biomedical AI | He et al., 2025 (main-text Ref. 28); scoping review of generative AI in time-varying biomedical data. | Yes at a methodological level. | Yes/partial across heterogeneous biomedical modalities. | Not ovarian-specific. | Transferred methodology. | Time-varying EHR, physiological, imaging, and omics data. | Clinical action thresholds were not the central classification. | Restricts transfer claims and verifies whether ovarian studies contain same-patient time and decision linkage. |
| General medical LLMs | Zhang et al., online 2025/2026 issue (Supplementary Ref. S9); scoping review of 415 healthcare LLM studies. | No ovarian trajectory validation; sequence processing and language generation were broad capabilities. | Partial. | Not addressed. | General LLM evidence. | Clinical text, EHR, education, communication, and research tasks. | No ovarian-specific validated action thresholds. | Constrains LLMs to interface functions under human oversight and separates language quality from clinical-management impact. |

Interpretation: “Longitudinal AI” requires explicit within-patient use of order, intervals, time-varying covariates, missingness, latent state, or risk updating—not merely a sequence-capable architecture. “Action thresholds” are prespecified actions evaluated with calibration, false-positive burden, net benefit, clinician override, or prospective impact. Main-text reference numbers correspond to the revised manuscript containing 102 references. Two authors independently performed the representative-study verification and classification; disagreements were resolved by consensus.

**Supplementary Table S2. Evidence matrix of representative temporal studies**

| **Study** | **Population** | **Design** | **Repeated measurements** | **Actual temporal input** | **Evidence category** | **T class** | **Outcome** | **Validation** | **Clinical action** | **Limitation** |
| --- | --- | --- | --- | --- | --- | --- | --- | --- | --- | --- |
| Yang et al., 2024 (main-text Ref. 30) | 1,392 epithelial ovarian cancer patients | Single-center retrospective classification study | No patient-level sequence | Clinicopathological, surgical, chemotherapy, and laboratory variables; key laboratory data mainly from the week before surgery | Direct ovarian AI evidence | T0 | Platinum-resistant recurrence classification | Five-fold internal cross-validation only | None predefined | Static baseline/short-window model; no within-patient biomarker sequence or external validation. |
| Li et al., 2020 (Ref. 31) | Patients with epithelial ovarian carcinoma | Retrospective biomarker association study | Yes | CA-125 dynamic-change summaries, including decline-related features | Ovarian longitudinal biological evidence | T1 | Progression-free survival | Single-cohort multivariable analysis | No predefined action | Serial observations were compressed into summary statistics; not an AI time-series model. |
| Xu et al., 2012 (Ref. 32) | 28,038 PLCO participants with at least two CA-125 screens; 72 incident ovarian cancers | Screening-cohort prediction study | Yes | CA-125 velocity with intervals between screening tests | Ovarian longitudinal biological/prediction evidence | T1 | Incident ovarian cancer | Internal discrimination and calibration assessment | Screening classification only | Screening, not post-treatment recurrence; cannot be directly extrapolated to recurrence monitoring. |
| Zeng et al., 2017 (Ref. 33) | Advanced epithelial ovarian cancer after interval debulking surgery | Retrospective prognostic association study | Yes | CA-125 nadir level and time to nadir | Ovarian longitudinal biological evidence | T1 | Survival | Single-cohort multivariable analysis | No predefined action | Nadir summarizes serial data; no explicit sequence model or decision threshold. |
| Chang et al., 2016 (Ref. 48) | Women followed after ovarian-cancer treatment | Joint longitudinal-event modeling study | Yes | Repeated CA-125 linked to recurrence time through a joint model | Direct ovarian temporal-model evidence (statistical) | T2 | Dynamic recurrence prediction | Internal model assessment | Potential risk updating; no validated action rule | Direct T2 candidate, but external validation and prospective clinical-impact evidence are limited. |
| MRC OV05/EORTC 55955, 2010 (Ref. 9) | 529 randomized patients in complete remission after first-line treatment | Multicenter randomized trial of early versus delayed treatment | Yes | Serial CA-125 surveillance with disclosure and treatment initiation after a defined rise | Ovarian decision-time clinical evidence (non-AI benchmark) | T3-relevant benchmark; not an AI model | Overall survival and quality of life | Randomized prospective evaluation | Treatment initiation after CA-125 rise | Earlier treatment did not improve overall survival; earlier detection is not equivalent to clinical benefit. |
| Yu et al., 2020 (Ref. 39) | Serous ovarian carcinoma pathology cohorts | Retrospective computational-pathology study | No | Single diagnostic/resection whole-slide image | Direct ovarian AI evidence | T0 | Histology and platinum-response-related prediction | Retrospective cohort testing; no temporal validation | None predefined | Static pathology evidence; not longitudinal imaging or recurrence-trajectory tracking. |
| Nguyen et al., 2023/2024 (Ref. 41) | Ovarian cancer patients with pathology and clinical data | Retrospective multimodal modeling | No | Single-time-point WSI features plus clinical variables | Direct ovarian AI evidence | T0 | Treatment-effectiveness prediction | Retrospective internal/held-out evaluation | No prespecified action threshold | Static multimodal evidence; temporal order and treatment-response updating were not modeled. |
| Wei et al., 2019 (Ref. 43) | Advanced high-grade serous ovarian cancer across centers | Multicenter retrospective radiomics study | No | Preoperative contrast-enhanced CT radiomics | Direct ovarian AI evidence | T0 | Recurrence/PFS risk stratification | Multicenter retrospective validation | Risk stratification only | Single preoperative scan; no serial imaging or dynamic risk update. |
| Zheng et al., 2022 (Ref. 44) | High-grade serous ovarian cancer | Retrospective CT deep-learning study | No | Preoperative CT representation | Direct ovarian AI evidence | T0 | Overall survival | Retrospective train/test evaluation | Risk stratification only | Single-time-point imaging. |
| Wang and Lu, 2021 (Ref. 45) | Advanced high-grade serous ovarian cancer | Retrospective PET/CT radiomics study | No | Baseline PET and CT radiomic features | Direct ovarian AI evidence | T0 | Progression-free survival | Single-cohort retrospective evaluation | Risk stratification only | Static PET/CT; not longitudinal multimodal imaging. |
| Lei et al., 2022 (Ref. 46) | Epithelial ovarian cancer | Retrospective MRI deep-learning study | No | Pretreatment MRI | Direct ovarian AI evidence | T0 | Platinum sensitivity/resistance | Retrospective internal testing | No predefined treatment action | Single-time-point MRI and no prospective treatment-impact evaluation. |
| Xu et al., 2019 (Ref. 47) | Non-small-cell lung cancer patients with serial CT | Retrospective serial-imaging model | Yes | Ordered pretreatment and follow-up CT images | Transferred methodological evidence | T2 | Treatment response and survival | Retrospective validation in lung cancer | Potential follow-up risk updating | Not ovarian cancer; supports method transfer only, not direct ovarian validation. |
| Koivuaho et al., 2019 (Ref. 49) | Birth cohort followed to age 46, including women with PCOS | Longitudinal cohort analysis | Yes | Repeated BMI trajectory and age at adiposity rebound | Ovarian longitudinal biological evidence | T1 | Adult PCOS diagnosis and obesity phenotype | Cohort association analysis | No model-linked action | Defines life-course biology; does not demonstrate deployed longitudinal AI. |
| Ayonrinde et al., 2016 (Ref. 50) | Adolescents with NAFLD/MASLD and/or PCOS | Life-stage cohort phenotype comparison | No repeated within-woman sequence for the reported comparison | Adolescent life-stage and comorbidity status | Ovarian longitudinal biological/context evidence | T0 | Adverse metabolic phenotype | Observational cohort comparison | No model-linked action | Supports stage-specific biology, not a patient-level trajectory model. |
| Tehrani et al., 2025 (Ref. 51) | Women with PCOS and controls under long-term follow-up | Functional data analysis of insulin-resistance trajectories | Yes | Repeated insulin-resistance measures modeled as functions over time | Ovarian longitudinal biological evidence | T2 | Trajectory differences and BMI modification | Single-cohort longitudinal analysis | No clinical action threshold | Biological trajectory evidence; no AI-driven dynamic-management trial. |
| de Kat et al., 2016 (Ref. 53) | Population-based women with serial AMH | Longitudinal cohort modeling | Yes | Repeated AMH across age with nonlinear population/mixed modeling | Ovarian longitudinal biological evidence | T2 | Population mean AMH decline and heterogeneity | Internal cohort estimation | No clinical action | Population-average patterns do not yield precise individual milestones; assay and measurement uncertainty remain. |
| de Kat et al., 2019 (Ref. 8) | 2,434 premenopausal women; 6,699 AMH samples over up to 20 years | Prospective cohort with longitudinal modeling and time-varying Cox prediction | Yes | Multiple AMH measurements and estimated decline pattern | Ovarian longitudinal biological evidence | T2 | Menopause and early-menopause prediction | Internal cohort model assessment | Potential counseling; no validated action rule | Decline rate did not improve prediction for most ages and early-menopause risk was underestimated in younger women. |
| Ramezani Tehrani et al., 2020 (Ref. 56) | 959 women with three AMH measurements | Longitudinal cohort prediction study | Yes | Annual AMH decline rate added to prediction models | Ovarian longitudinal biological evidence | T1 | Age at menopause | Internal discrimination assessment | Potential counseling only | Incremental performance was cohort-, assay-, age-, and model-dependent; no external action validation. |
| Miao et al., 2025 (Ref. 55) | Women with fundus images, AMH, and reproductive-history data | Cross-sectional/associational biomarker study | No within-woman repeated retinal-age modeling | AI-derived retinal age gap measured once and associated with reproductive-aging markers/events | Direct ovarian-associated AI evidence | T0 | Association with AMH and reproductive-aging phenotypes | Association analyses; no longitudinal impact study | None predefined | Exploratory cross-organ biomarker; not a longitudinal multimodal trajectory. |
| Xu et al., 2025, OvaRePred (Ref. 36) | 15,241 ART cycles for training and 14,498 later cycles for temporal testing | Single-center retrospective ART-cycle model | No long-term repeated AMH within the same woman | Age quadratic term and AMH cubic term predict POR; probability mapped to a population curve | Direct ovarian AI evidence | T0 | POR prediction and endocrine-age mapping | Same-center temporal split; ART population only | Potential risk communication | Trajectory-inspired population mapping; no individual decline rate; milestone projections are hypothesis-generating. |
| Kong et al., 2025 (Ref. 34) | 13,788 GnRH-agonist long-protocol IVF/ICSI cycles | Single-center retrospective deep-learning prescription study | Yes, within stimulation cycles | Ordered daily hormones, follicle measurements, prior doses, and stimulation day | Direct ovarian AI evidence | T2 | Daily FSH prescription prediction | Retrospective internal train/test evaluation | Clinician-facing prescription prediction | Historical prescriptions are behavior labels, not optimal-treatment labels; no prospective outcome benefit. |
| Ata et al., 2011 (Ref. 58) | 100 women undergoing controlled ovarian stimulation for IVF | Prospective comparison of automated and manual follicle monitoring | Repeated scans occurred during stimulation, but the automation target was per-scan measurement | Automated follicle count and measurement from three-dimensional ultrasound | Direct ovarian measurement-automation evidence | T0 measurement automation | Agreement/reproducibility of follicle monitoring | Prospective within-cohort method comparison | Measurement standardization | Measurement automation is not dose selection, explicit temporal prediction, or decision-linked treatment optimization. |
| Canon et al., 2024 (Ref. 35) | 291 prospective treatment-arm cycles across two centers with clinician acceptance/override | Multicenter prospective historical-control decision-support study | Yes, cycle-level data and decision points | Model recommendations for starting dose and trigger with clinician override | Direct ovarian AI evidence | Early T3 candidate | Mature oocytes, total FSH, safety, and process outcomes | Prospective multicenter evaluation | Clinician may accept, modify, or reject recommendations | Mature-oocyte and total-FSH differences were not statistically significant; larger concurrent trials and live-birth outcomes are needed. |
| Liu et al., 2026 (Ref. 97) | 200 standardized ART cases used to evaluate generated reasoning chains | Blinded comparative synthetic-reasoning evaluation | No patient-management time series | Prompt strategy and generated clinical-reasoning content | Direct ovarian AI evidence—synthetic reasoning evaluation | Not a patient temporal model | Expert-rated reasoning quality | Blinded senior-expert ratings compared with GPT-4o judging | No patient action or autonomous decision | Selective few-shot improved expert scores, but the AI judge did not reliably separate strategies; not a management system. |

Abbreviations: AFC, antral follicle count; AI, artificial intelligence; AMH, anti-Müllerian hormone; ART, assisted reproductive technology; CA-125, cancer antigen 125; EHR, electronic health record; FSH, follicle-stimulating hormone; ICSI, intracytoplasmic sperm injection; IVF, in vitro fertilization; LLM, large language model; MRI, magnetic resonance imaging; NAFLD/MASLD, non-alcoholic/metabolic dysfunction-associated steatotic liver disease; PCOS, polycystic ovary syndrome; PET/CT, positron-emission tomography/computed tomography; PFS, progression-free survival; POI, premature ovarian insufficiency; POR, poor ovarian response; T0-T3, temporal-information and decision-linkage classes defined in the main manuscript; WSI, whole-slide image. A study labeled “not a patient temporal model” does not estimate a patient-level ovarian trajectory or time-updated clinical risk.

**Supplementary-only review references**

S1. Breen J, Allen K, Zucker K, Adusumilli P, Scarsbrook A, Hall G, et al. Artificial intelligence in ovarian cancer histopathology: a systematic review. NPJ Precis Oncol. 2023;7:83. doi:10.1038/s41698-023-00432-6.

S2. Mitchell S, et al. Artificial intelligence in ultrasound diagnoses of ovarian cancer: a systematic review and meta-analysis. Cancers (Basel). 2024;16(2):422. doi:10.3390/cancers16020422.

S3. Ghaderzadeh M, Garavand A, Salehnasab C. Artificial intelligence in polycystic ovary syndrome: a systematic review of diagnostic and predictive applications. BMC Med Inform Decis Mak. 2025;25:427. doi:10.1186/s12911-025-03255-6.

S4. Kim JH. Current state of artificial intelligence applications in assisted reproductive technology: a narrative review. Clin Exp Reprod Med. 2025. doi:10.5653/cerm.2024.07710.

S5. Kakkar P, Gupta S, Paschopoulou KI, Paschopoulos I, Paschopoulos I, Siafaka V, et al. The integration of artificial intelligence in assisted reproduction: a comprehensive review. Front Reprod Health. 2025;7:1520919. doi:10.3389/frph.2025.1520919.

S6. Li W, et al. The potential of large language models in the field of infertility: a systematic review. J Assist Reprod Genet. 2025;42. doi:10.1007/s10815-025-03679-z.

S7. Carrasco-Ribelles LA, Llanes-Jurado J, Gallego-Moll C, Cabrera-Bean M, Monteagudo-Zaragoza M, Violán C, et al. Prediction models using artificial intelligence and longitudinal data from electronic health records: a systematic methodological review. J Am Med Inform Assoc. 2023;30(12):2072-2082. doi:10.1093/jamia/ocad168.

S8. Amirahmadi A, Ohlsson M, Etminani K. Deep learning prediction models based on EHR trajectories: a systematic review. J Biomed Inform. 2023;144:104430. doi:10.1016/j.jbi.2023.104430.

S9. Zhang Z, Momeni Nezhad MJ, Bagher Hosseini SM, Zolnour A, Zonour Z, Hosseini SM, et al. Advancing healthcare with large language models: a scoping review of applications and future directions. Int J Med Inform. 2026;208:106231. doi:10.1016/j.ijmedinf.2025.106231.
